# Symmetry Breaking by Surface Blocking: Synthesis of Bimorphic Silver Nanoparticles, Nanoscale Fishes and Apples

**DOI:** 10.1038/srep32561

**Published:** 2016-09-08

**Authors:** Nicole Cathcart, Vladimir Kitaev

**Affiliations:** 1 Department of Chemistry and Biochemistry, Wilfrid Laurier University, 75 University Avenue W, Waterloo, Ontario, Canada N2L 3C5

## Abstract

A powerful approach to augment the diversity of well-defined metal nanoparticle (MNP) morphologies, essential for MNP advanced applications, is symmetry breaking combined with seeded growth. Utilizing this approach enabled the formation of bimorphic silver nanoparticles (bi-AgNPs) consisting of two shapes linked by one regrowth point. Bi-AgNPs were formed by using an adsorbing polymer, poly(acrylic acid), PAA, to block the surface of a decahedral AgNP seed and restricting growth of new silver to a single nucleation point. First, we have realized 2-D growth of platelets attached to decahedra producing nanoscale shapes reminiscent of apples, fishes, mushrooms and kites. 1-D bimorphic growth of rods (with chloride) and 3-D bimorphic growth of cubes and bipyramids (with bromide) were achieved by using halides to induce preferential (100) stabilization over (111) of platelets. Furthermore, the universality of the formation of bimorphic nanoparticles was demonstrated by using different seeds. Bi-AgNPs exhibit strong SERS enhancement due to regular cavities at the necks. Overall, the reported approach to symmetry breaking and bimorphic nanoparticle growth offers a powerful methodology for nanoscale shape design.

Metal nanoparticles (MNPs) are advantageous for diverse applications including plasmonics^1^,^2^, catalysis^3^,^4^, sensing^5^,^6^ and medicine^7^,^8^. Understanding size and shape selection^9^,^10^ in MNPs is essential to fully capitalize on the advantageous nanoscale properties of MNPs^11^,^12^. Synthetic preparation of well-defined MNP morphologies is currently a bottleneck for MNP applications^13^. Seeded growth is an established approach to achieve nanoscale morphologies with increasing complexity^14^,^15^. Seeded growth requires limiting new nucleation events, which can be achieved by the slow addition of metal precursor^16^ or by utilizing ligands for complexation and selective binding^17^. To control the NP shape in seeded regrowth, surface-binding species can block specific NP facets by selective adsorption^18^. In particular, halides were found to play a crucial role in the shape-selective synthesis of AuNPs^19^ and AgNPs^20^,^21^.

Formation of bimorphic nanoparticles (bi-NPs) requires symmetry breaking that is postulated to be promoted by the high driving forces of reduction^22^, which can be realized through maintaining the high ratio of reducing agent to metal^23^,^24^. Symmetry breaking driven by the strain energy of growing NPs has been reported for Cu deposition onto Au seeds^25^ and for binary MNPs made from cubic Pt seeds^26^. Preparation of diverse nanostructures in bimorphic regrowth has been described by Tsuji *et al.*, where cubes, bipyramids, and platelets were formed from nanorods^27^. Bimorphic growth aims at combining two different shapes, similar to Janus particles^28^,^29^. Such nanoparticles are currently prepared through anisotropic branching^30^, which is widely applicable but limited in shape selectivity. Overall, general approaches for symmetry breaking and realization of bi-NPs consisting of two well-defined parts remain largely unexplored.

Herein we describe symmetry breaking resulting in bimorphic AgNPs with two well-defined constituent parts achieved through controlled blocking of the growing surface of AgNP seeds by poly(acrylic acid). In particular, controlled nucleation at the surface of decahedral AgNPs yielded single-point growth leading to size- and shape-selected bimorphic AgNPs. Different modes of bimorphic growth have been realized using halides. For 2-D growth of platelets attached to decahedra, we have elucidated conditions required for the symmetry breaking and shape selection and demonstrated that growth pathways can be judiciously controlled to engineer diverse complex nanoscale morphologies.

## Results and Discussions

**Figure 1:**
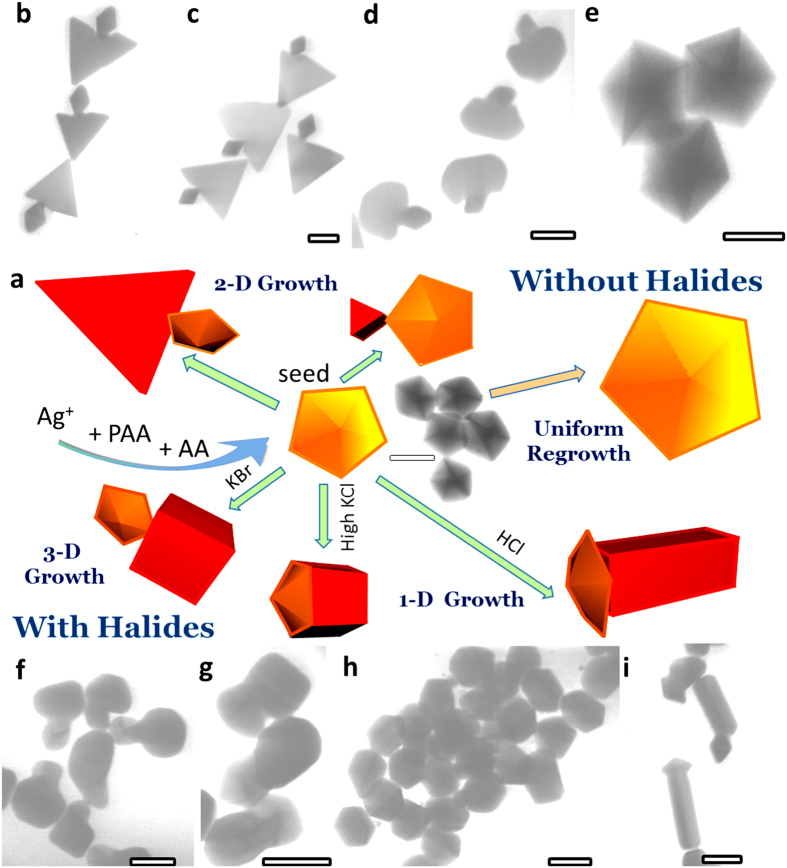
(**a)** Schematics of different pathways of bi-AgNP formation illustrating 1-D, 2-D and 3-D growth in the system. (**b**–**i)** transmission electron microscopy (TEM) images of (**b**–**d)** representative 2-D bi-AgNP morphologies, (**b**,**c)** optimal preparation: 0.13 mM PAA 450 K, 0.04 mM AgDeNP seeds, 0.27 mM ascorbic acid, and 0.08 mM AgNO_3_, (**c)** low Ag, and **(e)** uniformly 3-D enlarged decahedra (high PAA). (**f–i)** representative bi-AgNPs prepared in presence of halides: (**f**,**g)** 3-D bi-AgNPs, (**f)** 75:1 Ag/KBr; (**g**) 38:1 Ag/KBr; (**h**) pentagonal rods with 1:12 Ag/KCl, (**i**) 1-D bi-AgNPs with 1:5 Ag/HCl. All ratios are molar. All scale bars are 50 nm. For detailed description of samples-see Supplementary Table S1.

Key features of bi-AgNP synthesis by seeded growth of decahedral AgNPs, AgDeNPs, (pentagonal bipyramids, J_13_) are summarized in Fig. 1. The symmetry breaking in regrowth of high-purity decahedra^31^,^32^ takes place upon the reduction of silver ions (AgNO_3_) by ascorbic acid (AA) with surface blocking by poly(acrylic acid) (PAA) giving rise to shape-selective deposition of new silver^22^,^23^. Several types of well-defined bi-AgNPs are attainable by controlling four main parameters, the most crucial of which is i) ***surface-blocking by an adsorbing polymer*** (PAA of different molecular weights at different concentrations); followed by ii) *the amount of new silver added*, iii) *reducing power* (AA concentration and pH of the reaction), and iv) *presence* *of* *shape-selective agents* (e.g. halides).

**Figure 2:**
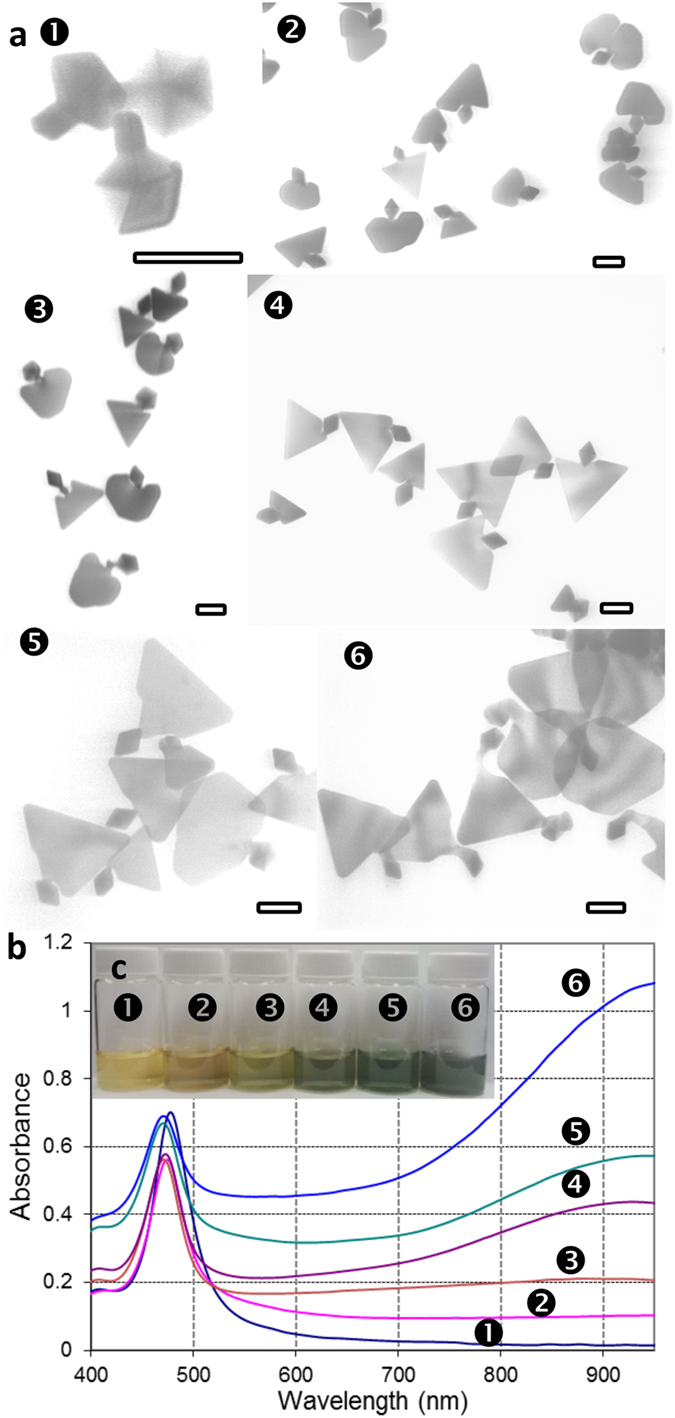
Development of 2-D bi-AgNP morphologies with different amounts of added silver (the percentage is given in brackets relative to silver present in decahedral seeds). (**a)** Representative TEM images; (**b)** UV-vis spectra; and (**c)** optical photographs of samples: ➊ 0.008 mM (20%), ➋ 0.016 mM (40%), ➌ 0.04 mM (100%), ➍ 0.05 mM (120%), ➎ 0.08 mM (200%) and ➏ 0.13 mM (320%). All scale bars are 50 nm.

**Figure 3:**
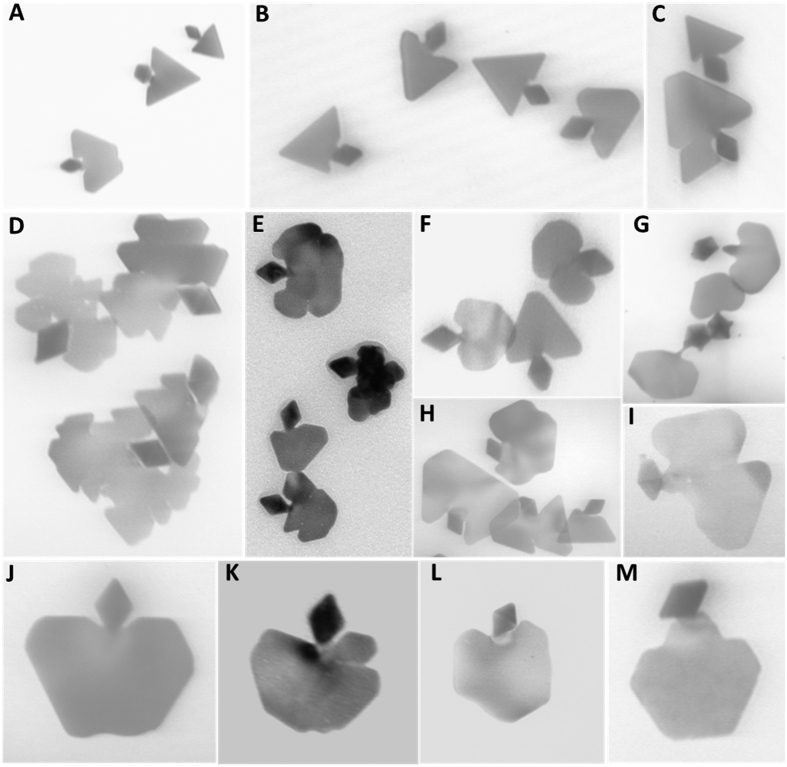
TEM images demonstrating diversity of attainable bi-AgNP shapes. The scale can be inferred from the largest dimension of the decahedral part of bi-AgNPs (a diamond shape in TEM side projection) being 41.5 ± 1.5 nm, reproducibly prepared by photochemical synthesis^32^.

AgNP morphologies attained in different regrowth conditions are shown in Fig. 1a. The main focus of this report is the uniform 2-D bimorphic growth of a platelet/prism onto a decahedral seed (Fig. 2 and Supplementary Fig. S1). Among other growth modes of decahedra, most straightforward is uniform 3-D regrowth (uniform decahedra enlargement) where new silver deposits uniformly onto all ten (111) facets^31^,^32^. In this report we have accessed 3-D regrowth of decahedra at low pH and in the presence of citrate at room temperature (Fig. 1e and Supplementary Fig. S2h). Several other regrowth modes can be accessed using halides: 1-D enlargement of decahedra resulting in pentagonal rods (Fig. 1a), mediated by chloride complexation and 3-D bimorphic growth of cubes and bipyramids from decahedral seeds (Fig. 1a and Supplementary Fig. S3) directed by bromide. Different pathways of the symmetry breaking in bi-AgNP growth demonstrated by this work (Figs 1 and 3) attest to the universality of the developed procedure. In particular, we have focused on 2-D bi-AgNPs to elucidate the role of synthetic parameters and to demonstrate control over shape selection.

Bi-AgNP formation requires a tight set of reaction conditions, in particular, the seed surface is appropriately blocked/protected so that nucleation can occur only in a single point. This nucleation point is one of the equivalent high energy vertices of the pentagonal rim in decahedra. The nucleation on the polymer-blocked surface is a limiting stage in the bimorph growth. Once the nucleation is initiated, growth of the bimorph proceeds rapidly through the formation of connecting ‘necks’ and subsequent more directional growth of the platelet part through their planar twinned defects^33^. Bi-AgNPs with two nucleated centers at different vertices (i.e. two platelets growing on the same decahedral seed) were not experimentally observed, even in minor quantities. The growth of the bimorph with the seed surface strongly blocked by an adsorbing polymer (PAA) results in high driving forces of reduction^22^ (per available surface) and is kinetically driven. Such growth proceeds with multiple twinning that is typical for the kinetically driven nanoparticle formation and is characteristic of the platelet growth^33^. Bi-AgNPs formed in optimized 2-D growth conditions feature platelets predominantly attached in a perpendicular orientation relative to the pentagonal planes of the decahedral seed (Figs 1b,c and 2, and Supplementary Figs S1 and S2a,b), as expected from the preferential growth at the vertices. At the same time, thin longer necks connecting platelets to decahedra (e.g. Supplementary Fig. S4b,c) were formed in conditions where the decahedral seeds were allowed to equilibrate with PAA prior to silver reduction. These long necks (along with high-magnification images shown in Supplementary Fig. S4a) clearly illustrate the point that there is no epitaxy and the crystalline plane orientation of the seed is not preserved in the regrowth at the heavily polymer-blocked surface.

Effects of major parameters in bi-AgNP formation are summarized in Figs 2 and S2 (and corresponding Table 1 and Supplementary Table S1). At higher PAA concentrations, the decahedra surface is completely blocked for the selective nucleation of the bimorphic growth and enlarged decahedra are a predominant product (Supplementary Fig. S2e). At lower PAA concentrations, the bimorphic growth is less-defined, so limited shape-control can be achieved (Supplementary Fig. S2f). The size of the platelet part can be controlled by the ratio of added silver to silver in AgDeNP seeds; increased added silver (300%, Fig. 2a6) resulted in larger platelets, and smaller growth was achieved with lower silver (20–40%, Figs 2a1 and 2). An effect of high AA concentration manifests in less-defined, chaotic reduction of silver resulting in rough AgNPs^34^ (Supplementary Fig. S2c); while at lower AA concentrations rounded etched decahedra form (Supplementary Fig. S2d). At higher pH, the secondary nucleation dominates due to the higher reducing power of AA (Supplementary Fig. S2g). At lower pH with the less reducing power and slower regrowth, enlarged decahedra are again the main regrowth product (Supplementary Fig. S2h) in absence of the symmetry breaking. Table 1 summarizes main bi-AgNP shapes described in this work and corresponding conditions for their preparation.

Blocking of the seed surface by PAA significantly restrains silver growth at the surface, effectively increasing the ratio of the reducing agent to reacting silver^35^ and thus creating higher driving forces similar to what was reported in the literature for breaking symmetry during MNP regrowth^22^,^23^. More details on the effect of PAA is provided in Supplementary (Supplementary Fig. S5 and its description). The equilibration time of the seed with PAA prior to the regrowth initiation (addition of silver nitrate and AA) was an important factor since kinetics of silver reduction plays a significant role in the formation of bi-AgNPs^12^. Formation of well-defined bi-AgNPs was attained at minimal equilibration times when AA and AgNO_3_ are added right after the addition of PAA to the decahedral seeds.

PAA molecular weight and the effect of other polymers have been investigated since PAA is the key reagent^36^ in bi-AgNP synthesis. Large M_W_ PAA (450,000) was found to be an optimal polymer for surface blocking. Lower M_W_ of PAA that we have tested (such as M_W_ of 1,800 and 5,100) gave similar but largely inferior results with respect to the uniformity of bi-AgNP growth and resulting size-dispersity (Supplementary Fig. S6b,c). More details are given in Supplementary Fig. S6 and its description. Citrate (or citric acid) in replacement of or in addition to PAA has also been explored since citrate is a commonly used charge-stabilizing agent for AgNPs by virtue of its multiple carboxylic groups. We were not able to find conditions of bi-AgNP synthesis using citric acid or citrate alone. At concentrations greater than 0.03 mM, citric acid tends to promote uniform 3-D growth of the decahedral seeds to larger decahedra (Supplementary Fig. S7a). Details on the effect of different acids are given in Supplementary Fig. S7 and its description.

For stable, reproducible regrowth with high shape yields, the optimal concentration of AgDeNP seeds is 0.03–0.04 mM, below this concentration, more irregular platelet growth occurs (Supplementary Fig. S8). With new silver added at a typical 200% molar ratio relative to silver in seeds, the total silver concentration of bi-AgNP dispersions is 0.1–0.15 mM (Fig. 2), typical for colloidal synthesis of metal NPs. At this range of concentrations it is possible to largely avoid aggregation and formation of doublets in the regrowth, as well as to minimize secondary nucleation that becomes a factor at lower seed concentrations. At ca. 300% of new silver (relative to the seeds), platelet parts can reach ca. 150–200 nm in width with typical thickness of 5–10 nm (Fig. 2a6), similar to platelet AgNPs prepared with citrate^37^,^38^. Experiments with lower amounts of added silver (Fig. 2a1) confirmed that the bi-AgNP growth is initiated predominantly at the vertices of the pentagonal plane of the decahedra as the locus of the sharpest protrusions and thus the highest surface energy points. The growth initially proceeds through disordered multiple planar twinned structures and subsequently resolve in favorable conditions into well-defined platelet parts (Fig. 2a3–6). The role of reducing conditions is described in detail in Supplementary Fig. S9. Overall, optimal AA concentration range was 0.25–0.7 mM at pH of 6–6.5. pH plays a dual role in bi-AgNP synthesis: 1) due to the strongly pH-dependent reduction potential of ascorbic acid; 2) in AgNP stabilization, which is optimal in weakly basic conditions due to negative surface charge of stabilized AgNPs. Pure AgNP surface is positively charged due to silver ions present in the redox equilibrium. This positive charge is effectively reversed with common stabilizing anionic ligands, such as citrate or ionized poly(acrylic acid). The negative charge of the stabilizing ligands is neutralized at acidic pH due to protonation of carboxylic groups. Experiments with pH variation are shown in Supplementary Fig. S10.

Halides have a rich legacy in shape control of silver nanoparticles^39^, e.g. bromide has been shown to be effective in transformation of Ag planar twinned morphologies into 3-D cubes and bipyramids by stabilizing (100) planes^38^. Bromide has been shown experimentally and computationally to strongly coordinate to silver and to drive selective facet stabilization in silver and gold nanoparticles^40^. In our work, the presence of bromide enables the transformation of 2-D bimorphic growth into 3-D by arresting platelet growth, first making them smaller and thicker (Fig. 1a) and then transforming them into rounded cubes and bipyramids, as shown in Fig. 1f,g and Supplementary Fig. S11. Rounded cubes attached to decahedra can be observed at Ag/Br molar ratios as low as 138:1 (Supplementary Fig. S11a), also confirmed by UV-vis spectroscopy (Supplementary Fig. S12). In the presence of bromide, the overall shape of bi-AgNPs is less defined due to etching with bromide and interference with reducing conditions by the bromide effect on silver reduction potential. Chloride exhibits a more complex and subtle effect on bi-AgNP formation. First, more than 100 times higher chloride concentrations were required to reach an effect comparable to bromide due to the appreciably larger K_sp_ of AgCl vs. AgBr. Stabilization of (100) similar to bromide was evident (Fig. 1h and Supplementary Fig. S13). 1-D growth of decahedra to short pentagonal rods was noticeably promoted at higher Cl/Ag ratio (>12:1) (Fig. 1h and Supplementary Fig. S13d), where the reduction is slower due to chloride both complexing silver ions and blocking the seed surface. 1-D pentagonal growth of decahedra can be distinguished from 1-D bimorphic growth, where the rods are nucleated in a single point at the surface of AgDeNPs, and are not necessarily pentagonal (Fig. 1a). The addition of smaller chloride amounts (Ag/Cl of 20–50) had a weak positive effect on the formation of well-defined triangular platelet parts of bi-AgNPs. 1-D bimorphic growth was observed when HCl was used to adjust pH (Fig. 1i and Supplementary Fig. S14). This growth mode takes place in mild reducing conditions where the deposition of new silver is directed by pentagonal twinning disclination with subsequent rebuilding of (100) planes^41^, and protonation of PAA (Fig. 1i). Compared to bromide and chloride, iodide has a strong arresting effect on the development of bi-AgNPs (see Supplementary Fig. S15 and description therein).

The diversity of attainable bi-AgNP morphologies is represented in Fig. 3. Many bi-AgNP nanoshapes are reminiscent of fish (Fig. 3A–C), birds, mushrooms, kites and butterflies. In particularly, we aimed to produce nanoscale analogues of apple-like shapes (Fig. 3J–M), especially apples with a bite taken out (Fig. 3K,L), as being culturally significant. Such bi-AgNPs with additional symmetry breaking could be prepared in slightly more etching conditions relative to optimal. A complementary nanoscale pear-like bi-AgNP is shown in Fig. 3M. See also supplementary Fig. S19 for selected coloured images.

Further bimorph diversity can be realized with different seed particles for the regrowth. To demonstrate such possibility, we have used silver platelets/prisms prepared using our previously reported procedure^38^. Using 2-D regrowth conditions, formation of the second platelet part is achieved by nucleation and growth at high-energy vertices to yield platelet-platelet bimorphic AgNPs (Supplementary Fig. S16). Therefore any suitable seed particles are expected to be successfully used in the bimorphic regrowth with suitable polymer blocking of the seed surface.

Formation of Au coating and shells^42^,^43^ of bi-AgNPs was tested with HAuCl_4_ addition. Even at lower amounts of gold relative to silver in bi-AgNPs, such as 5 mol.%, the gold deposition is highly granular, with the nucleation and growth only in selected spots on the surface (Supplementary Fig. S17a,b). At ca. 20 mol.% of gold added, the resulting nanostructures become rough due to silver etching (Supplementary Fig. S17c).

Bi-AgNPs show excellent SERS enhancement by virtue of their packing that creates semi-regular voids due to incompatibility with close packed lattices^31^. Using 5,5′-dithiobis (2-nitro-benzoic acid) (DTNB) as a probe molecule and 785 nm laser, 10^−16^ moles/cm^2^ could be easily detected with dry bi-AgNP films as substrates (Supplementary Fig. S18), and DTNB detection limit can be estimated as ca. 5 × 10^−16^ moles/cm^2^. The signal enhancement relative to pure DTNB is 7 × 10^9^ vs. 4 × 10^9^ of AgDeNPs. Noticeably higher SERS enhancement relative to decahedra may be attributed to the resonant SERS enhancement due to LSPR absorption at 785 nm from the platelet parts of bi-AgNPs, and to the cavity at the junction of the two bimorph parts, where the field enhancement is expected to be the highest. As highly non-centrosymmetric particles, bi-AgNPs can be also beneficial for plasmonic applications^44^.

In summary, we have demonstrated that by controllably blocking the surface of the seed AgNPs with an adsorbing polymer (PAA), several modes of seeded growth from uniform (111) enlargement of decahedra to symmetry breaking with a single point nucleation result in diverse bimorphic morphologies. Universality of the formation of bimorphic nanoparticles using different seeds is also demonstrated. Bi-AgNPs exhibit strong SERS enhancement and are promising as a SERS substrate. Demonstrated approach to symmetry breaking and resulting bimorphic particles open new perspectives in nanoscale shape design.

## Methods

AgDeNP seeds were prepared as described by Murshid *et al.*^32^. AgDeNPs (prepared 2–20 days prior to bi-AgNP synthesis) were first concentrated 10 times (from 0.13 mM to 1.3 mM Ag). Representative amounts and total molarities in the final preparation (in brackets) for well-defined 2-D bi-AgNPs (shown in Fig. S10 for pH = 6) were as follows: 6 mL of water, 40 μL of 0.02 M PAA (M_w_ = 450,000, 0.13 mM by monomer), 200 μL of concentrated AgDeNPs (0.04 mM), 35 μL of 0.05 M ascorbic acid (0.27 mM), and 100 μL of 0.005 M AgNO_3_ (0.08 mM) combined in a 20 ml vial upon magnetic stirring. Upon addition of AgNO_3_, the reaction color changed from yellow-orange to greenish-bluish grey. See Supplementary Table S1 for specific conditions of synthesis of other bi-AgNP morphologies. More details on reagents, procedures of bi-AgNP preparation and characterization are provided in Supplementary.

## Additional Information

**How to cite this article**: Cathcart, N. and Kitaev, V. Symmetry Breaking by Surface Blocking: Synthesis of Bimorphic Silver Nanoparticles, Nanoscale Fishes and Apples. *Sci. Rep.* **6**, 32561; doi: 10.1038/srep32561 (2016).

## Supplementary Material

Supplementary Information

## Figures and Tables

**Table 1:**
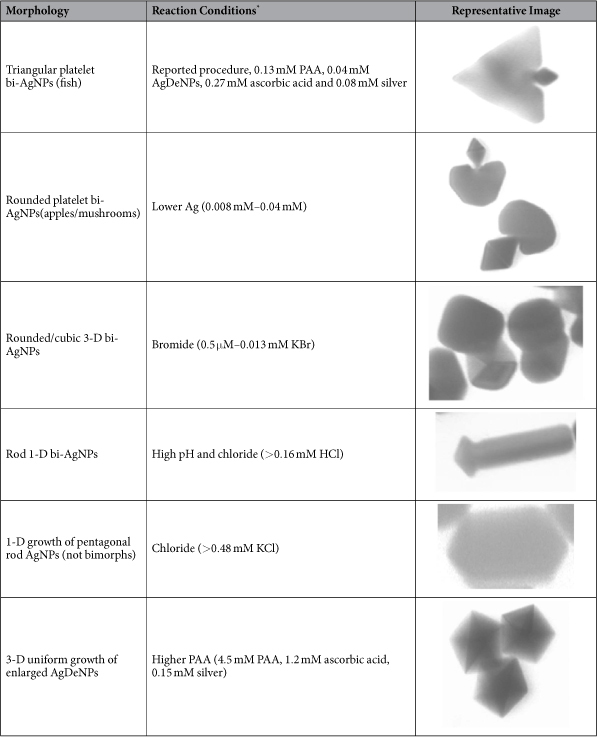
Summary of experimental conditions for the formation of well-defined bimorphic AgNP shapes.

^*^Concentrations are the same as reported in Methods for 2-D growth of the bimorphs with triangular platelets, unless otherwise stated.
