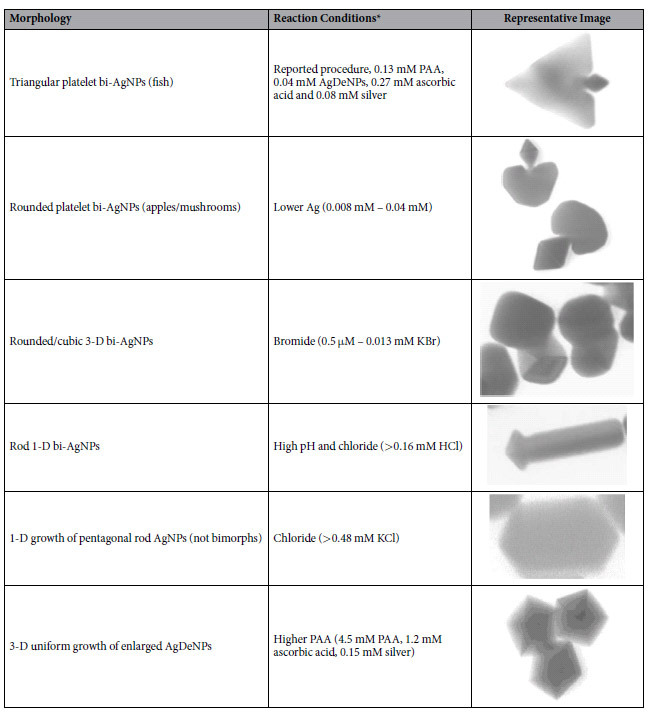# Erratum: Symmetry Breaking by Surface Blocking: Synthesis of Bimorphic Silver Nanoparticles, Nanoscale Fishes and Apples

**DOI:** 10.1038/srep37203

**Published:** 2016-11-22

**Authors:** Nicole Cathcart, Vladimir Kitaev

Scientific Reports 6: Article number: 3256110.1038/srep32561; published online: 09 08 2016; updated: 11 22 2016

In the HTML version of this Article, Table 1 has been omitted. Table 1 appears below.

## Figures and Tables

**Table 1:**