# Supplementary material for: Symmetry Breaking by Surface Blocking: Synthesis of Bimorphic Silver Nanoparticles, Nanoscale Fishes and Apples
Source: Sci Rep. 2016 Sep 8;6:32561. doi: 10.1038/srep32561 (PMC5015045; doi:10.1038/srep32561)
Supplement: Supplementary Information [file srep32561-s1.pdf]

Supplementary Information

Symmetry Breaking by Surface Blocking: Synthesis of Bimorphic Silver Nanoparticles: Nanoscale Fishes and Apples

Nicole Cathcart and Vladimir Kitaev

Experimental details

Reagents. Silver nitrate (99.99%), sodium citrate tribasic dihydrate (99.5%), arginine (98%), sodium borohydride (≥99%), hydrogen peroxide (30-32 wt%, 99.999% trace metal basis, potassium stannate inhibitor), poly(acrylic acid) (PAA, avg. M_w= 1,800), poly(acrylic acid) (PAA, avg. M_w = 450,000), poly (acrylic acid sodium salt) (PAA, avg. M_w= 5,100), poly (acrylamide co-acrylic acid), partial sodium salt (PA co-AA, avg. M_W = 200,000 , 20 % acrylamide), poly (N-vinylpyrrolidone-co-2-dimethylaminoethyl methacrylate) 19+ wt. % solution in water (PVP-co-DMAEMA, avg. M_w = 1,000,000, Batch #14030EO), poly (sodium 4-styrenesulfonate) (PSS, avg. M_w = 70,000), L-ascorbic acid, (99+%), 5,5'-dithiobis (2-nitro-benzoic acid) (99%), potassium chloride (99%), potassium bromide (99%), potassium iodide (99%), potassium hydroxide (99.99%), nitric acid (65 wt.%, ACS grade), hydrochloric acid (37 wt.%, semiconductor grade), all supplied by Aldrich; and poly (N-vinylpyrrolidone) (PVP, avg. M_W = 40,000) supplied by Caledon Chemicals (Caledon, Canada) were used as received. High purity deionized water (> 18.2 MΩ·cm) was produced using Millipore A10 Milli-Q.

3-D growth of Decahedra with PAA. The procedure is the same as the synthesis of bimorphic NPs except for the higher PAA concentration used. In a typical preparation corresponding to the sample shown in Fig. S3E, 6 mL of water, 150 µL of 0.2 M PAA 450 K (4.5 mM), 200 µL of concentrated decahedra with 1.3 mM Ag (0.04 mM), 160 µL of 0.05 M ascorbic acid (1.2 mM), and 200 µL of 0.005 M $AgNO_3$ (0.15 mM). Numbers in brackets are total molarities in the final preparation. After the addition of silver nitrate, the reaction mixture remains visually unchanged for 20-30 minutes, before reduction of silver on the decahedral seed surface becomes noticeable by a red-shift in the LSPR. The regrowth process was typically completed within 12-18 hours.

Preparation of SERS substrates. SERS substrates were prepared by concentrating 1.5 mL of bi-AgNP to 20-50 µL dispersions using centrifugation. The resulting total silver concentration in concentrated bi-AgNP samples was 10-20 mM. 2-10 µL of this concentrated dispersion was transferred onto a quartz

slide by a pipette and spread to a size of 1 cm by 0.6 cm. The sample was dried in an oven at 55-60 °C for 5-10 minutes, and then used for SERS measurement without further treatment. 1-5 µL of dilute 5,5'-dithiobis(2-nitrobenzoic acid) in water (with addition of THF at higher concentrations) was dispensed directly onto the dried bi-AgNPs and then dried in an oven for 1-2 minutes.

Instrumentation. Electron microscopy (EM) imaging was done using Hitachi S-5200 with a carbon-coated formvar grid (EMS Corp.). UV-vis spectra were acquired with either an Ocean Optics QE-65000 fiber-optic UV-vis spectrometer or Cary 50Bio UV-vis spectrophotometer. Raman spectra were recorded using R-3000QE fibre-optic Raman spectrometer equipped with 290 mW laser at 785 nm (RSI). Centrifugation was performed using either VWR Clinical 100 or Thermo Scientific Legend Micro 21 centrifuges.

Table S1. Summary of synthetic conditions for bi-AgNP samples presented in Figures 1 and S2.

Ag^+ Added (mM)	Decahedra Seed (mM)	Polymer, MW, (mM)	Ascorbic Acid (mM)	Other Parameters	Sample #	Corresponding Figure Image/Morphology
0.069	0.039	PAA*, 1.8 K, 0.063	0.938	Tricitrate, 0.038 mM	NJ523	1b
0.070	0.040	PANa*, 5 K, 0.002	0.278		NN283	1c
0.018	0.040	PAA, 450 K, 0.096	0.438		NO487	1d
0.143	0.036	PAA, 1.8 K, 0.347	1.627	Citric Acid, 0.578 mM	NO77	1e
0.069	0.039	PAA, 450 K, 0.126	0.276	KBr, 0.002 mM	NQ396	1f
0.063	0.039	PAA, 450 K, 0.125	0.274	KBr, 0.006 mM	NT625	1g
0.062	0.039	PAA, 450 K, 0.124	0.272	KCl, 1.24 mM	NT648	1h
0.063	0.039	PAA, 450 K, 0.126	0.275	HCl, 0.154 mM	NT606	1i
0.069	0.035	PAA, 1.8 K, 0.063	0.629	Tricitrate 0.31 mM	NH909	S2a

0.069	0.039	PAA, 1.8 K, 0.063	0.938	Tricitrate 0.39 mM	NJ523	S2b
0.017	0.039	PAA, 450 K, 0.063	1.255		NO624	S2c
0.069	0.039	PAA, 1.8 K, 0.25	0.039	AA added last	NH999	S2d
0.0556	0.032	PAA, 450 K, 3.788	1.010		NO690	S2e
0.070	0.040	PAA, 450 K, 0.015	0.278	KBr 0.11 μM	NP446	S2f
0.063	0.039	PAA, 450 K, 0.126	0.275	KOH 1.58 mM	NT517	S2g
0.132	0.038	PAA, 450 K, 4.506	1.201	HNO_3 0.15 mM	NP151	S2h
0.069	0.035	PAA, 450 K, 0.313	0.274		NO304	Lily pod (2D growth)
0.070	0.035	PANa, 5 K, 0.0016	0.278	NaOH 16 μM	NQ224	Fish (2D growth)
0.009	0.035	PAA, 450 K, 0.031	1.259		NO710	Small growth (2D growth)
0.068	0.035	PAA, 1.8 K, 0.248	0.776	AA* added last	NI7	Large decahedra (3D growth)

*PAA = poly (acrylic acid); PANa = poly (acrylic acid sodium salt); AA = ascorbic acid

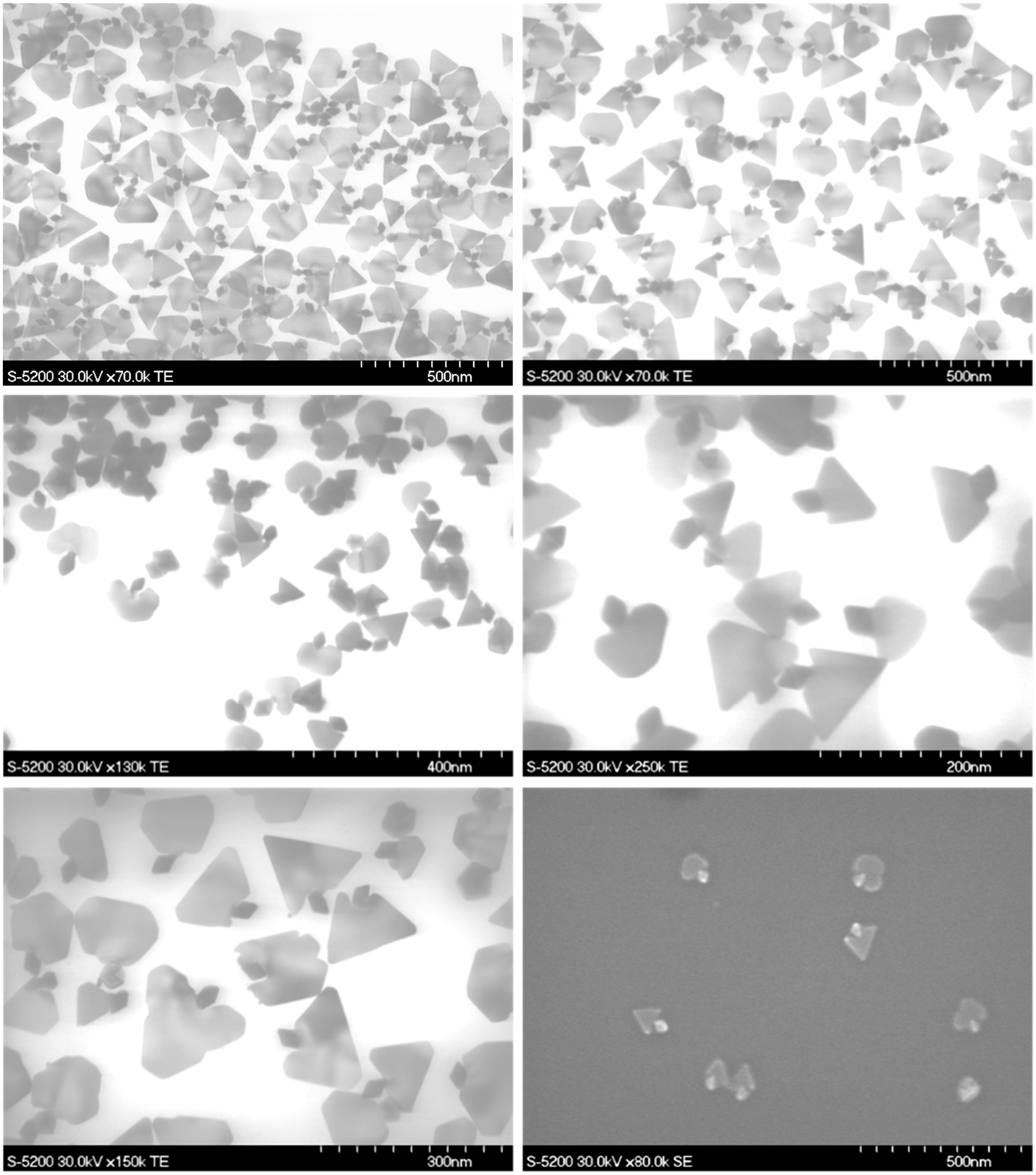

Figure S1. Representative electron microscopy (EM) images demonstrating high shape yield and consistent morphologies of 2-D bi-AgNPs prepared over the course of three years.

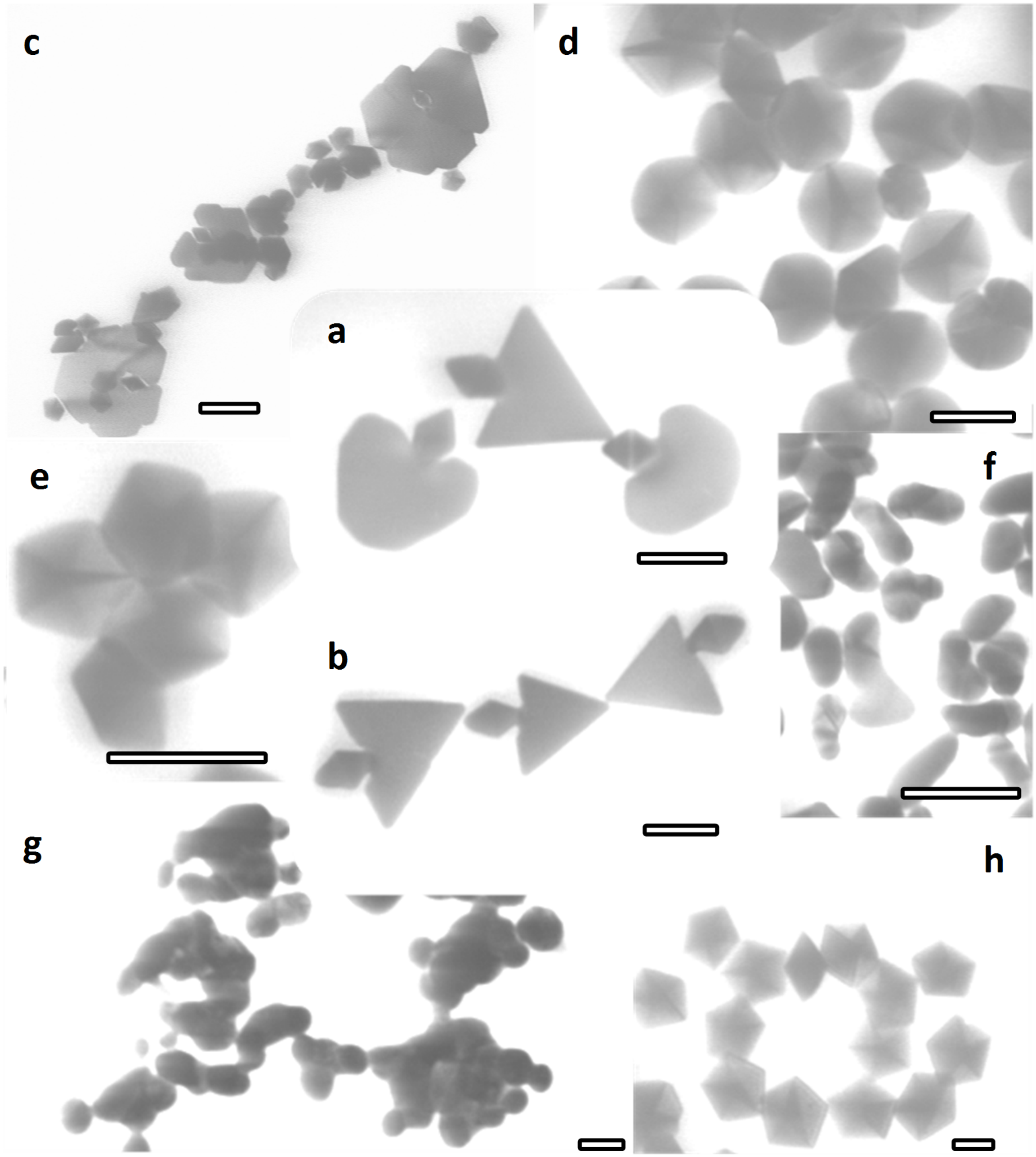

Figure S2. TEM images of key bimorphic morphologies prepared in optimized synthetic conditions: **a)** and **b**); and in synthetic conditions appreciably different from optimal: **c**) high ascorbic acid, **d**) low ascorbic acid, **e**) high PAA, **f**) low PAA, **g**) high pH and **h**) low pH. For detailed description of samples - see **Table S1**. Scale bars are 50 nm for **a)**, **b)**, **d)**, **g)** and **h)**; and 100 nm for **c)**, **e)** and **f)**.

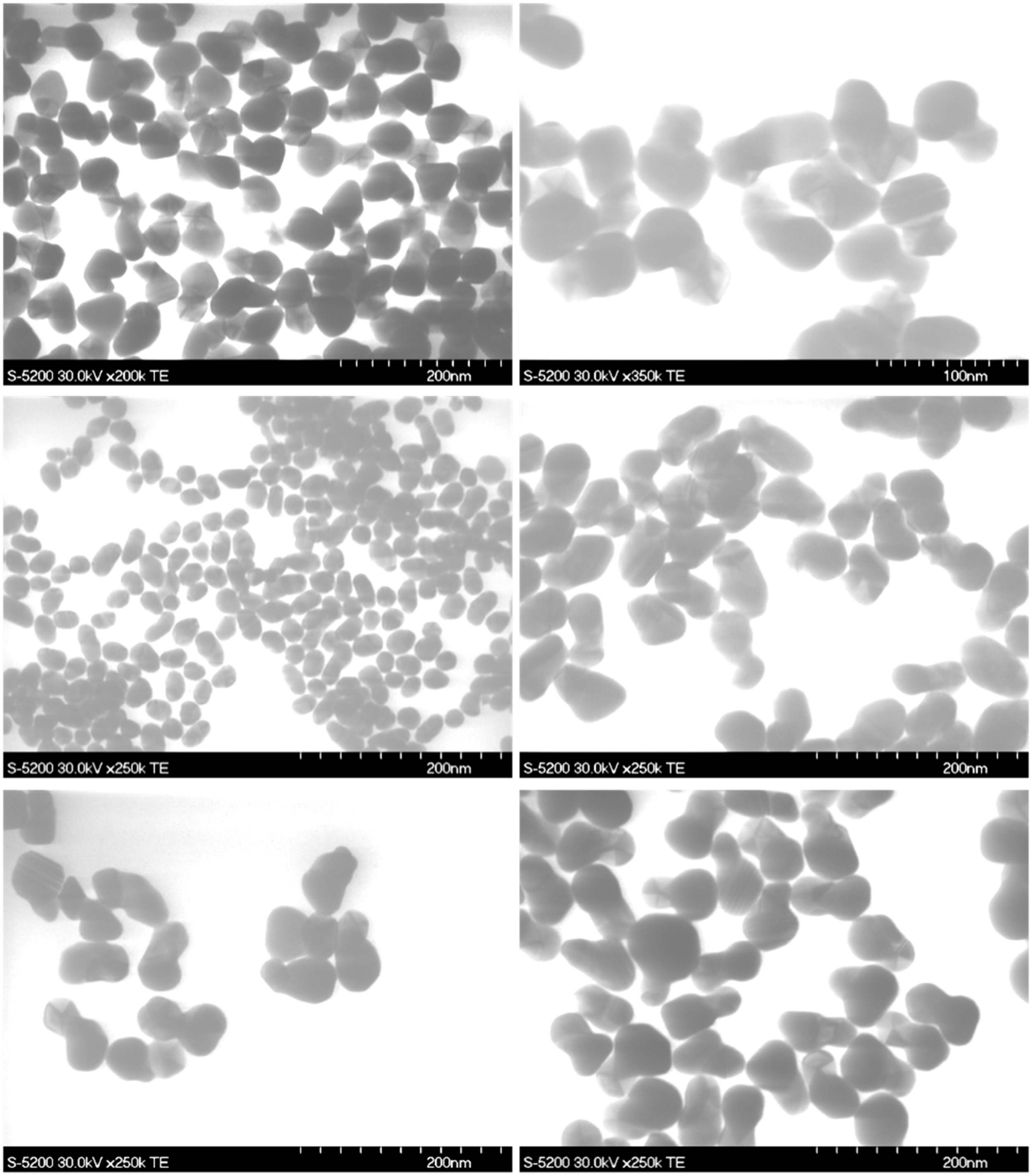

Figure S3. Representative transmission electron microscopy (TEM) images showing high shape yield of 3-D bi-AgNP regrowth.

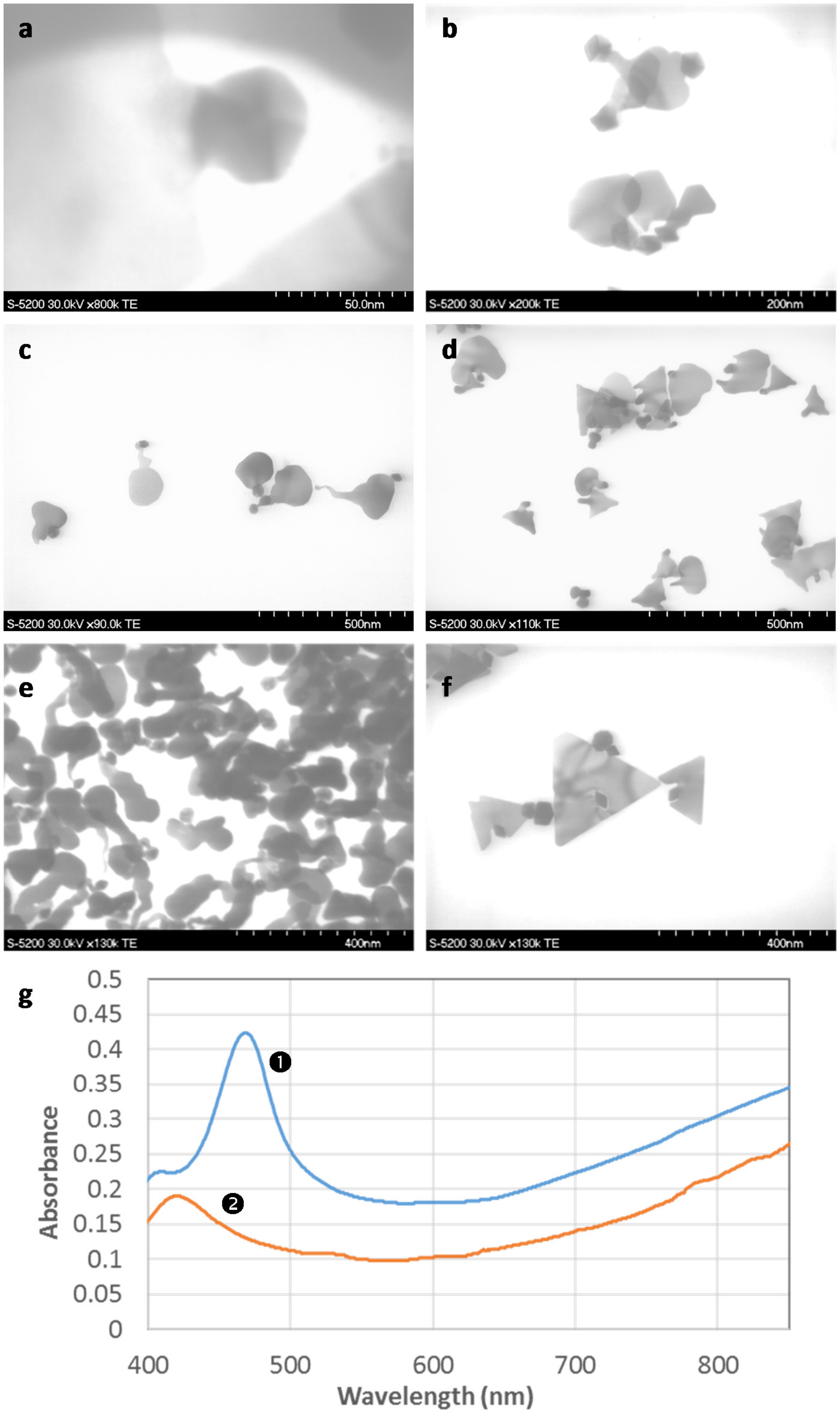

Figure S4. **a-f)** TEM images of irregular growth due to **a**) higher reagent concentration (25% less water), **b**) high PAA, **c**) equilibration between PAA and decahedra (delay in development), **d**) aging (6 days between preparation and imaging) **e**) high silver, **f**) high silver and ascorbic acid and **g)** UV-vis spectra of Fig. S4d aging ❶- immediately after preparation, ❷- 6 days after preparation (before imaging).

Thin necks of the bi-AgNPs shown in Figure S4c are likely due to the slower growth initiation and corresponding higher growth rates when silver starts to deposit onto the decahedra. This faster initial growth results in more disordered growth areas at the decahedra surface that subsequently become the necks. In addition, the decahedral part of bi-AgNPs gets more etched, which is evident from EM images of Fig. S4, as well as the blue shift of decahedra LSPR mode from 470-480 nm to 435-450nm (Fig. S4g). The effect of the equilibration with PAA is relatively subtle, so it was difficult to point out an appreciable difference between several minutes and several hours of equilibration. When higher AA concentrations were used together with high silver concentrations, newly generated metallic silver was deposited around the decahedral seeds in a less organized fashion with the rougher platelet-like structures growing with little to no neck or tethering (Fig. S4f).

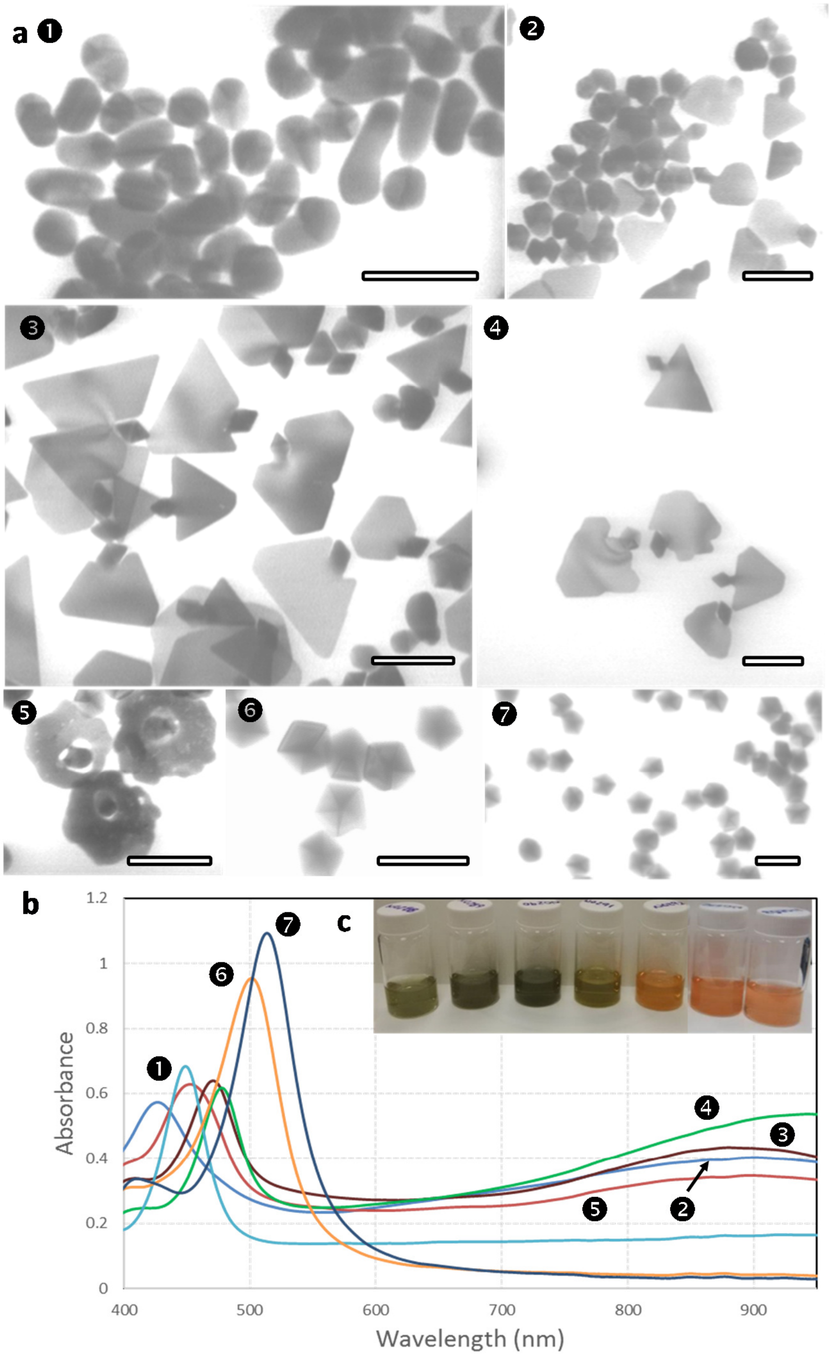

Figure S5. a) TEM images, **b)** UV-vis spectra and **c)** optical photographs of representative bi-AgNP samples prepared with increasing concentrations of poly(acrylic acid) (M_w =450,000): ❶ 0 mM, ❷ 0.03 mM, ❸ 0.12 mM, ❹ 0.25 mM, ❺ 0.76 mM, ❻ 1.1 mM and ❼ 2.2 mM. All scale bars are 100 nm.

In the absence of PAA and with typical AA concentrations, the decahedra regrowth is not well defined (Fig. S5a1), with Ag^+ depositing without preferential direction. At a higher but still less than optimal PAA concentration, the platelet formation starts to emerge but the bi-AgNP growth is still not well resolved (Fig. S5a2). At low PAA concentrations and higher AA concentrations with corresponding higher rates of silver reduction, new silver deposits around the periphery of decahedra leading to lily-pod morphologies (Fig. S8a) and decahedra encapsulation (Figs. S9a3 and S4f). At optimal PAA concentrations at 0.12-0.14 mM (ca. 1:1 molar ratio to total silver), well defined triangular platelets with the preservation of the decahedral part form (Fig. S5a3). At higher than optimal PAA concentrations, the decahedra surface becomes partially blocked to the extent that a significant portion of the decahedra seeds do not grow (Fig. S5a5). Consequently, the fast growth is initiated only on a fraction of decahedra seeds and proceeds in effectively less than optimal conditions – higher AA and silver concentration relative to the growing seeds. As a result, the silver deposition gets noticeably perturbed with the new silver depositing disorderly around the periphery of the decahedral seeds (Figs. S5a5 and S9a3) due to a larger effective ratio of new silver to the available growth surface in the seeds.

At even higher PAA concentrations (ca. 10-20 times higher than optimal, see Experimental), the surface of decahedral seeds becomes uniformly blocked to such an extent that the fast growth (5-10 seconds to minutes) is no longer possible and the deposition is switched to the slow (15-30 minutes to several hours) uniform 3-D growth of decahedra where (111) surfaces are homogeneously rebuilt (Figs. S5a6, S5a7). The transition between the 2-D bimorphic growth and 3-D decahedra enlargement is observed at PAA concentrations in a range of 0.8 to 1 mM (ca. 7-8 PAA/Ag molar ratio) and higher.

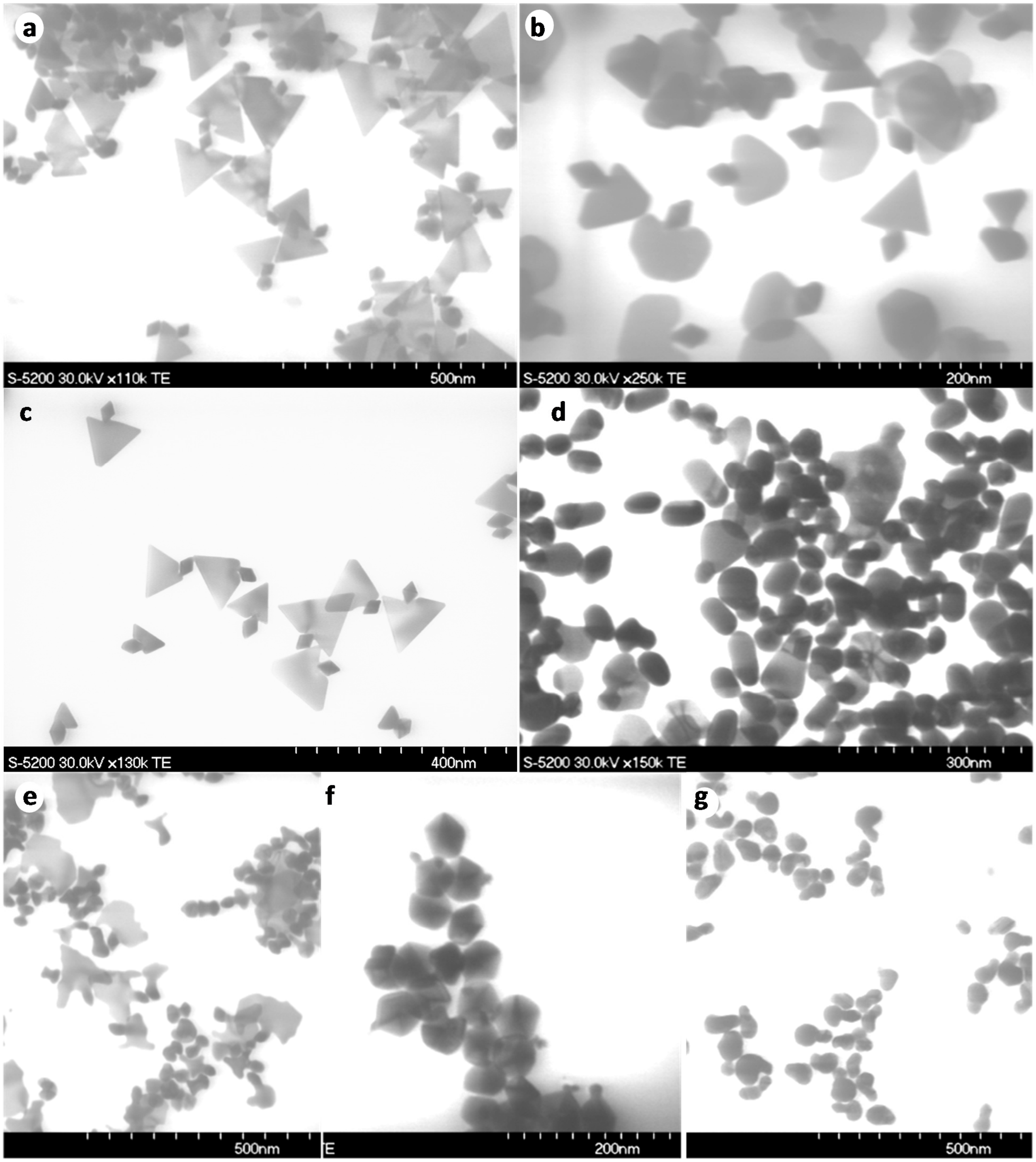

Figure S6. TEM images of bi-AgNP samples prepared using different polymers: **a**) poly(acrylic acid) 450 K, **b**) poly(acrylic acid) 1.8 K, **c**) poly(acrylic acid sodium salt) 5.1 K, **d**) poly(N-vinylpyrrolidone) 40 K, **e**) poly(acrylamide co-acrylic acid), partial sodium salt 200 K, **f**) poly(N-vinylpyrrolidone-co-2-dimethylaminoethyl methacrylate) 1,000 K, **g**) poly(sodium 4-styrenesulfonate) 70 K.

Poly(acrylic acid sodium salt) (PANa) with a MW of 5,100 produced noticeably well-defined triangular platelets in several samples, with more random attachment points to the decahedral part (Fig. S6c). A copolymer of acrylic acid with acrylamide was also explored (see Experimental). This copolymer supported growth of bi-AgNPs but with less-defined and more random platelet growth and less preserved decahedral parts of bi-AgNPs (Fig. S6e). Stabilization with poly(N-vinylpyrrolidone), PVP, and a copolymer of N-vinylpyrrolidone with dimethylaminoethyl methacrylate did not produce well-defined morphologies likely due to weak binding to the surface of AgNPs in absence of carboxylic groups (Figs. S6d,f). Poly(styrenesulfonate), PSS, provided weaker surface blocking of the decahedral seeds and the resulting bi-AgNPs becoming more rounded, with close attachment of the decahedra and platelet components (Fig. S6g).

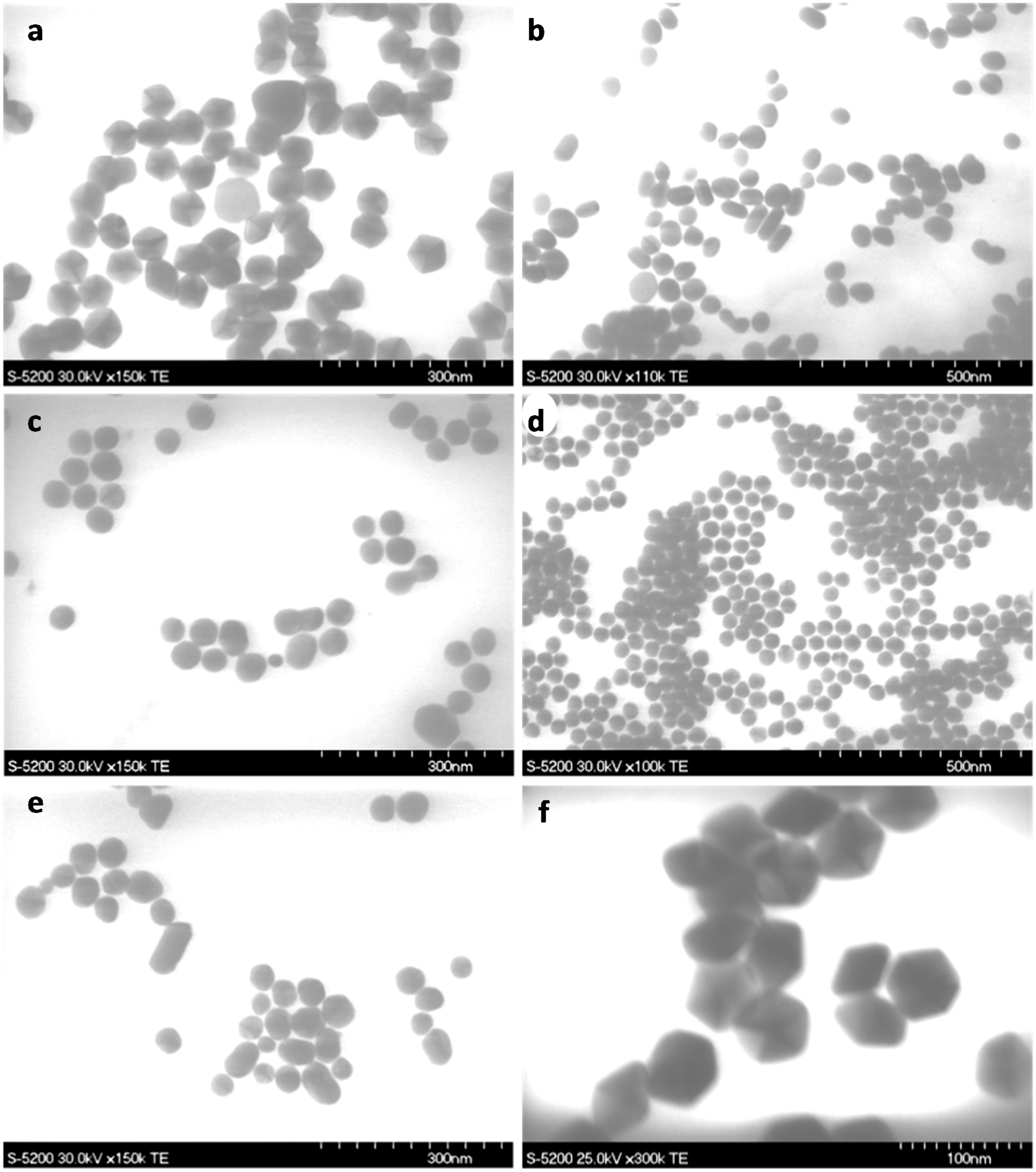

Figure S7. TEM images of samples prepared with various acids in replacement of PAA: **a**) citric acid, **b**) boric acid, **c**) nitric acid, **d**) trimesic acid, **e**) phosphoric acid, and **f**) tartaric acid.

At lower concentrations of citric acid and in the absence of PAA, the insufficient surface blocking and platelet stabilization result in uncontrolled growth. At the same time, citric acid or citrate could be used together with PAA, since they did not significantly affect bimorphic growth at concentrations up to 0.05

mM. These findings confirm the importance of long polymer chains of PAA in modulating the growth of AgNPs and the carboxylic groups playing a primary role in binding to the AgNP surface.

We have also conducted a series of experiments using several different acids in place of PAA to access their effect on bi-AgNP formation. As can be seen in Figure S7, all acids with exception of trimesic acid (Fig. S7d) etch the decahedra surface and yield either larger rounded decahedra or shapeless aggregated AgNPs. The preservation of decahedral shape with trimesic acid is due to the fact that it is a tricarboxylic acid similar to citric acid and it similarly stabilizes (111) planes in AgNPs.

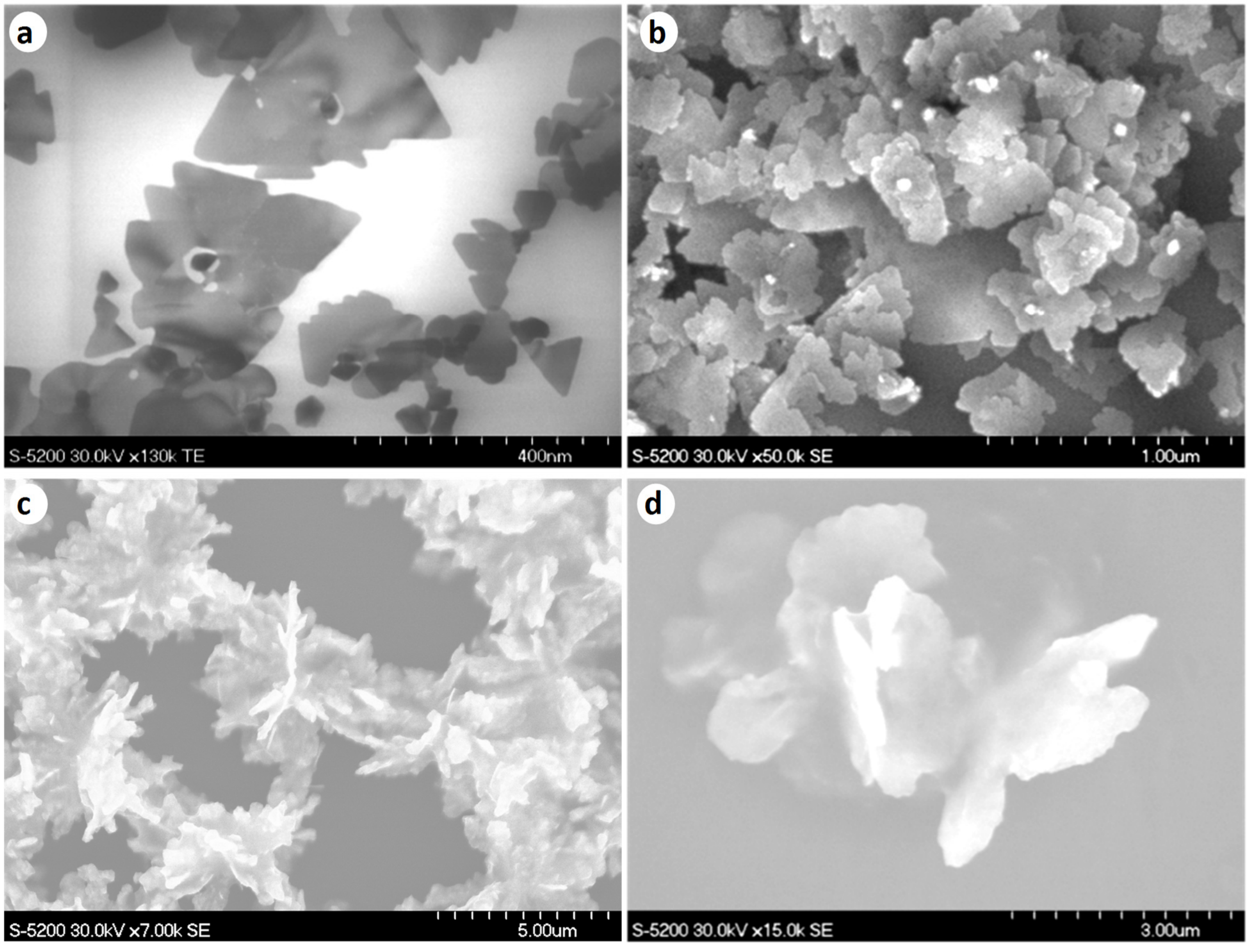

Figure S8. EM images (all SEM with the exception **a**), which is TEM) of bi-AgNP samples prepared with lower than optimal ratio of decahedral seeds to silver added in the regrowth: **a**) 1/2 compared to the optimal (See Experimental), **b**) 1/20, **c**) and **d**) no decahedral seeds.

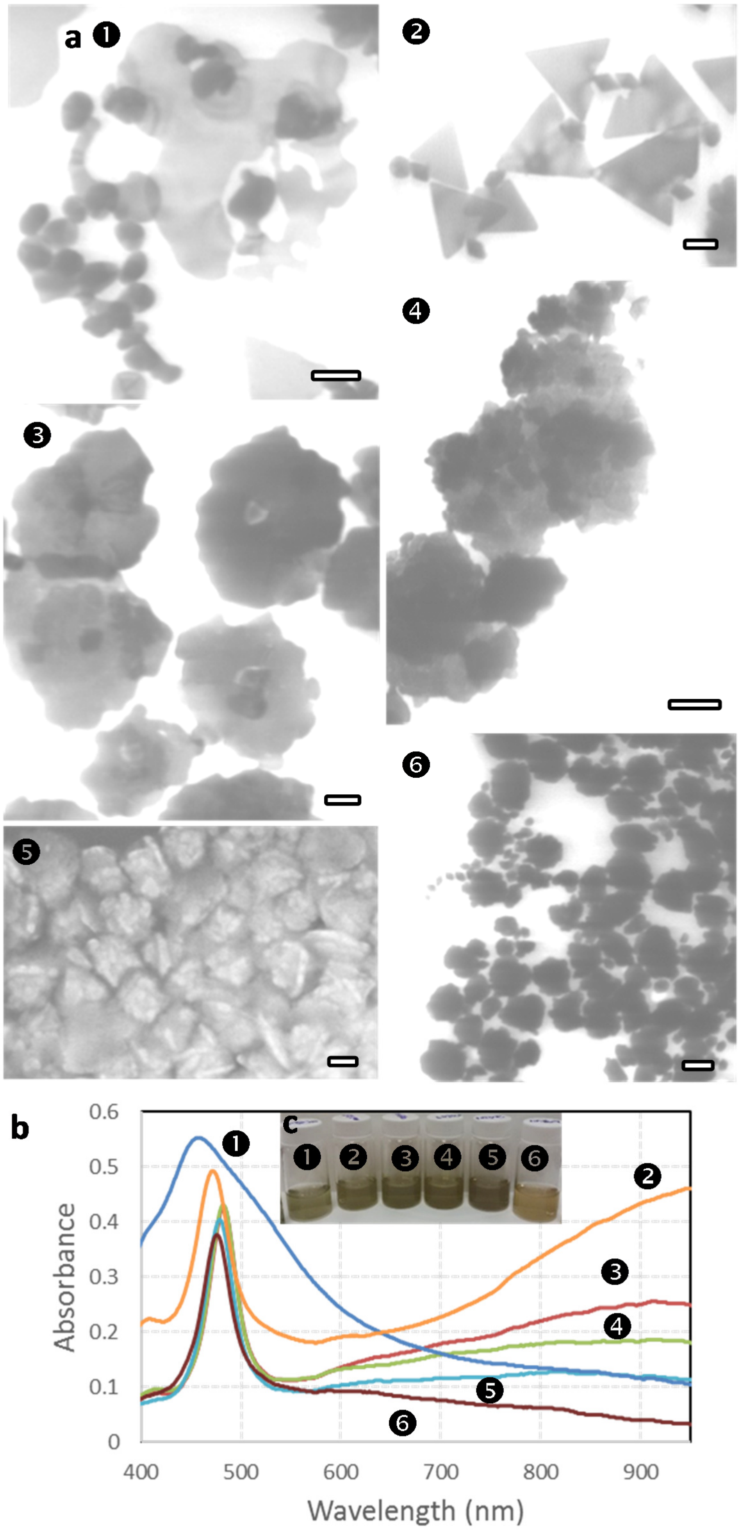

Figure S9. a) Representative EM (all TEM, except ❺ is SEM) images; **b)** UV-vis spectra; and **c)** optical photographs of samples prepared with increasing concentration of ascorbic acid: ❶ 0.08 mM, ❷ 0.3 mM, ❸ 0.7 mM, ❹ 1.4 mM, ❺ 2.8 mM, and ❻ 6.2 mM. Scale bars are 50 nm for ❶,❷ and ❸, and 100 nm for ❹,❺ and ❻.

The process of bi-AgNP formation involves the reduction of silver precursors, so the role of a reducing agent in the synthesis is important. In conditions of slow growth, e.g. photochemical transformations with citrate,[1] the decahedra enlargement in a 3-D growth mode is favoured. A commonly used reducing agent, sodium borohydride, is too powerful for bi-AgNP formation since it readily promotes secondary nucleation. Ascorbic acid proved to be optimal; first, by its moderate reducing strength and, second, because its reducing potential can be controlled (and thus fine-tuned) not only by concentration but also by pH, so that the reduction can be adjusted to proceed relatively fast but avoiding secondary nucleation. A common problem with the ascorbic acid is that it tends to induce faster growth with limited shape selection[2] (similar to what is shown in Figs. S9a3&4), so the reduction conditions need to be tightly controlled to produce well-defined uniform AgNP morphologies.

In a series of samples with varying AA concentration, shown in Figure S9, the prevailing trends can be readily seen. At lower AA concentrations, the excess of unreduced silver ions etches silver decahedra and the growth of planar twinned morphologies becomes less defined (Fig. S9a1). At higher than optimal AA concentration, the bimorphic growth becomes too rapid to lead to shape selection. The predominant observed morphologies are multiple platelets fused together, first around the rim of decahedra (Fig. S9a3) and then entirely around the seed particle resulting in 3-D aggregates (Figs. S9a4-6). A similar scenario is observed when less seed particles are used and fused platelets grow into large quasi-spherical structures (Fig. S8).

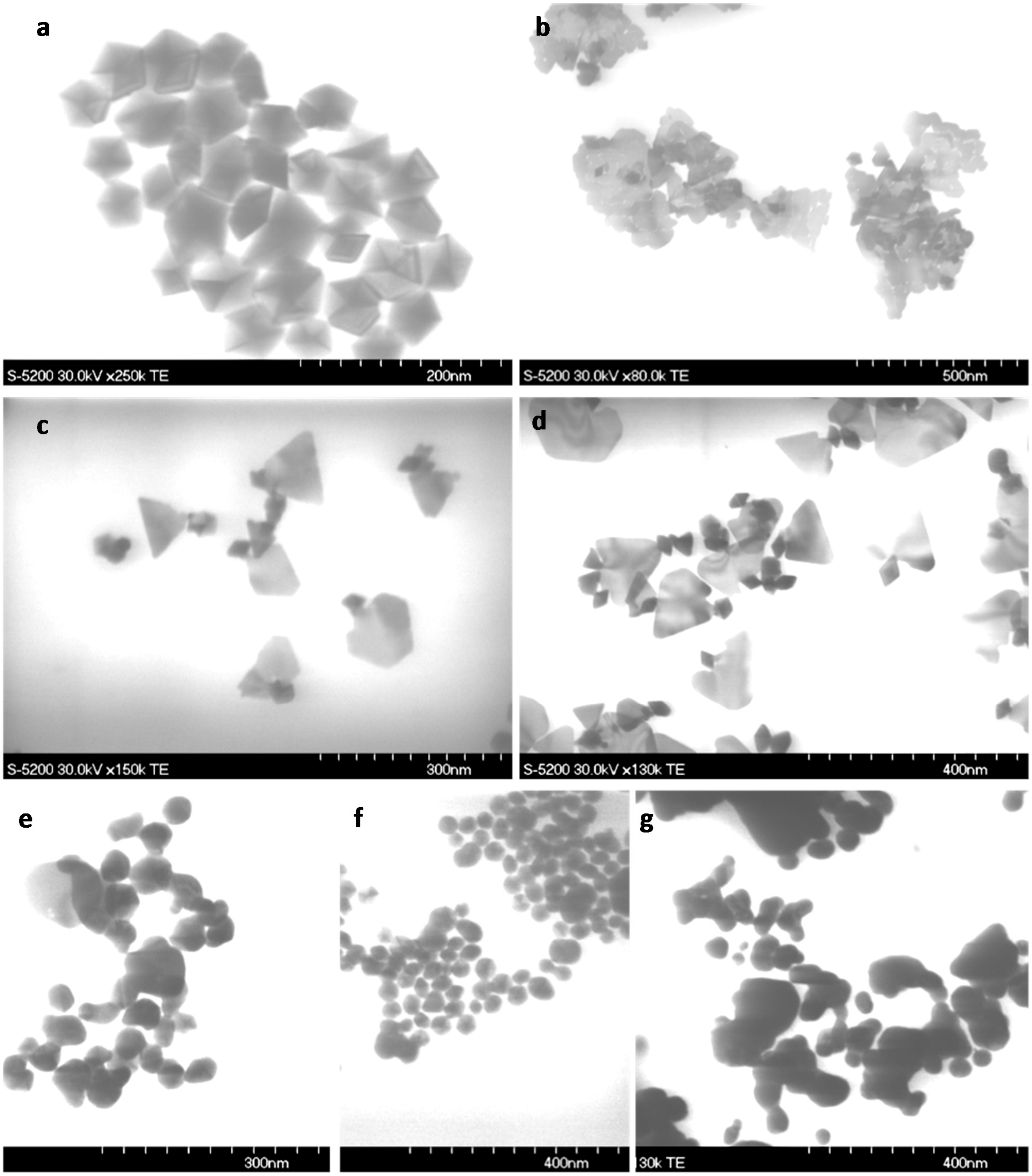

Figure S10. TEM images of bi-AgNP samples prepared at different pH: **a**) 5, **b**) 5.5, **c**) 6, **d**) 6.5, **e**) 7, **f**) 7.5 and **g**) 8.

In experiments with pH variation, the best bi-AgNPs are produced in a fairly narrow pH range of 6.0 to 6.5 (Fig. S10). At more acidic pH, AgNPs are expectedly etched, destabilized and aggregated. At higher

pH, it is likely that PAA becomes fully deionized and blocks the surface more effectively that results in more disturbed growth of bimorphic structures (Fig. S10). In addition, the higher reduction potential of ascorbate at higher pH may play a significant interfering role. At the same time, the effect of pH on the AA reduction potential can be potentially counteracted or fine-tuned by adjusting the concentration of ascorbic acid.

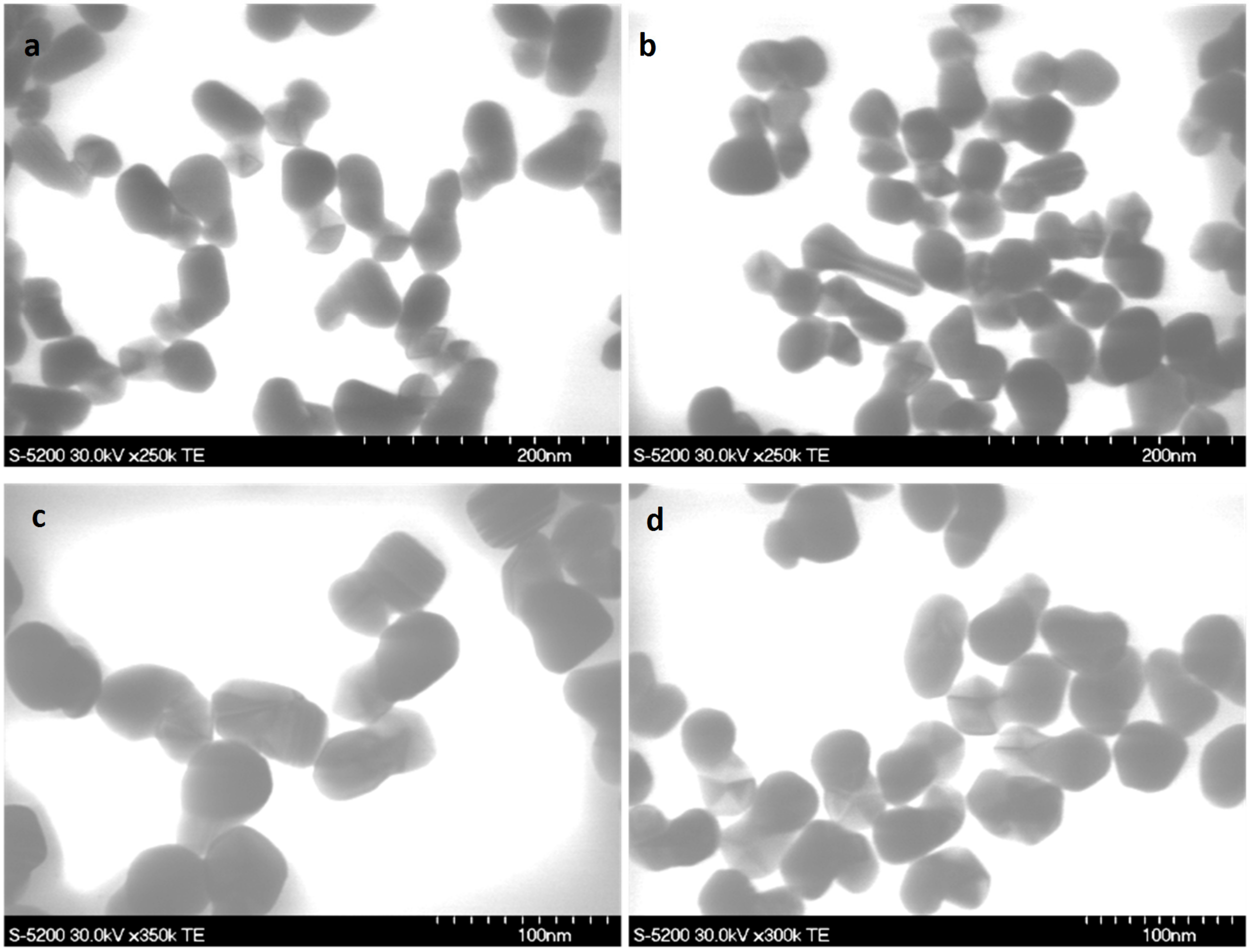

Figure S11. TEM images of bi-AgNP samples prepared using potassium bromide with Ag/Br molar ratio of **a**) 138:1, **b**) 25:1, **c**) 12:1 and **d**) 6:1.

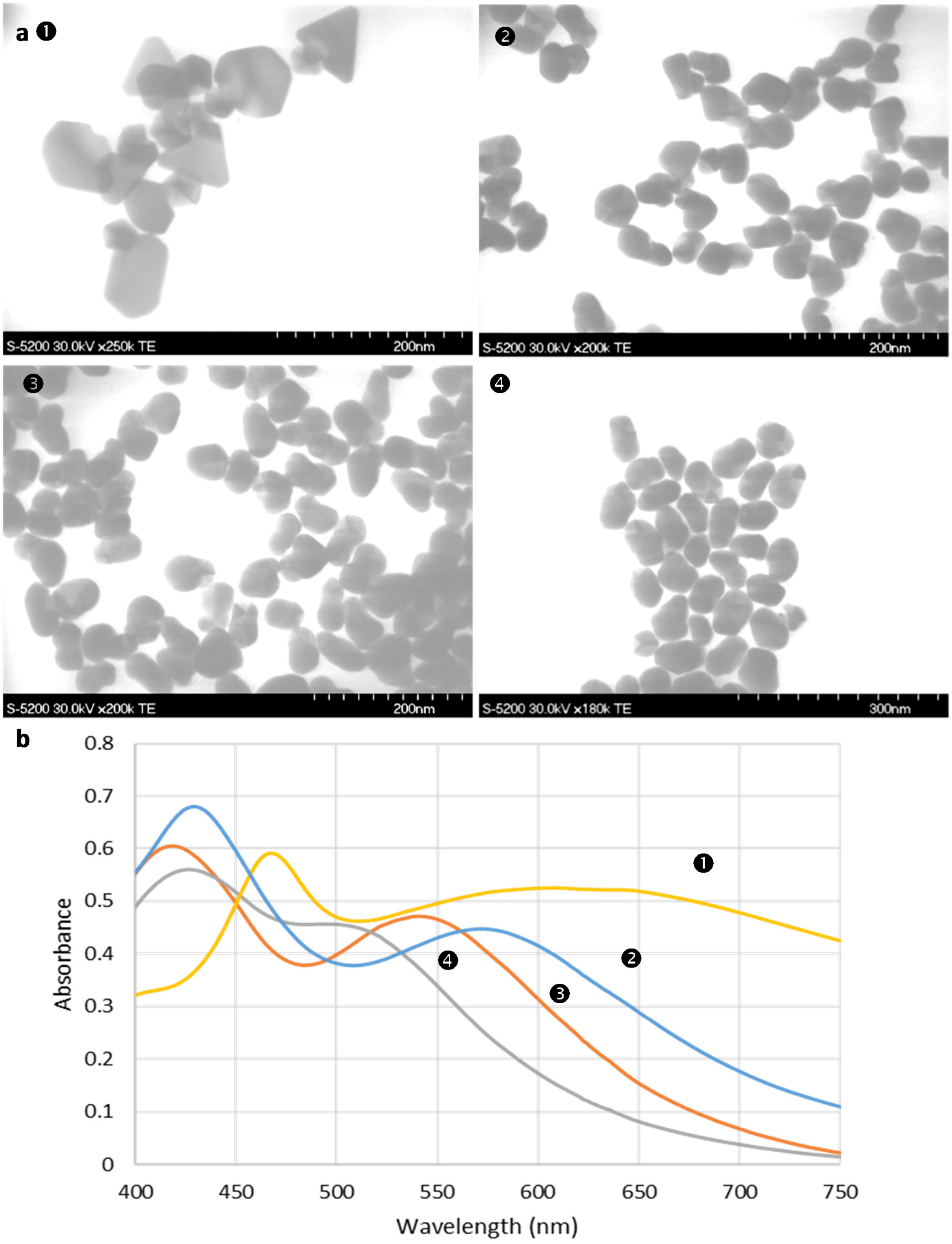

Figure S12. Development of 3-D bi-AgNPs morphologies with different amounts of bromide. **a)** Representative TEM images; and **b)** UV-vis spectra of samples with Ag/Br molar ratio of: ❶ 750, ❷ 75, ❸ 23, ❹ 7.5.

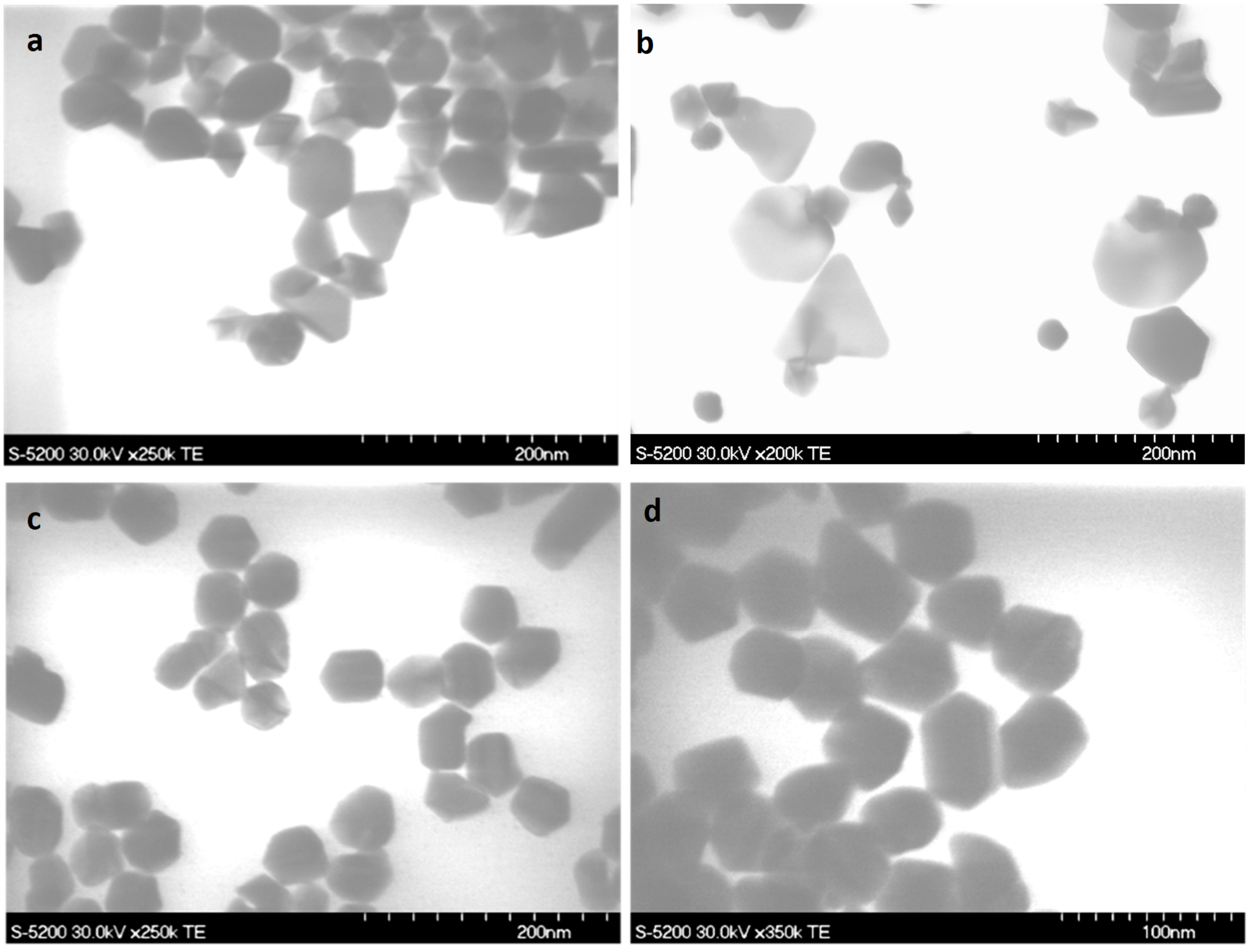

Figure S13. TEM images of bi-AgNP samples prepared using potassium chloride with Ag/Cl molar ratio of **a**) 16:1, **b**) 8:1, **c**) 1:6 and **d**) 1:12.

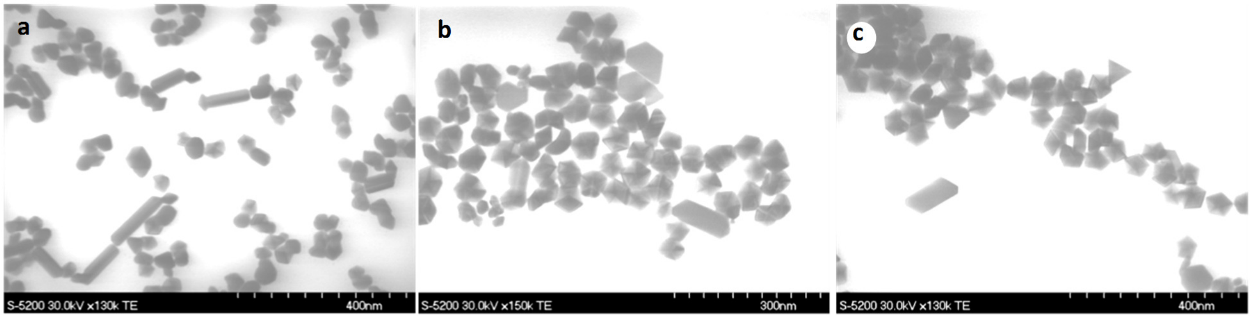

Figure S14. TEM images of bi-AgNP samples prepared using HCl with Ag/Cl molar ratio of **a**) 1:2, **b**) 1:4 and **c**) 1:8.

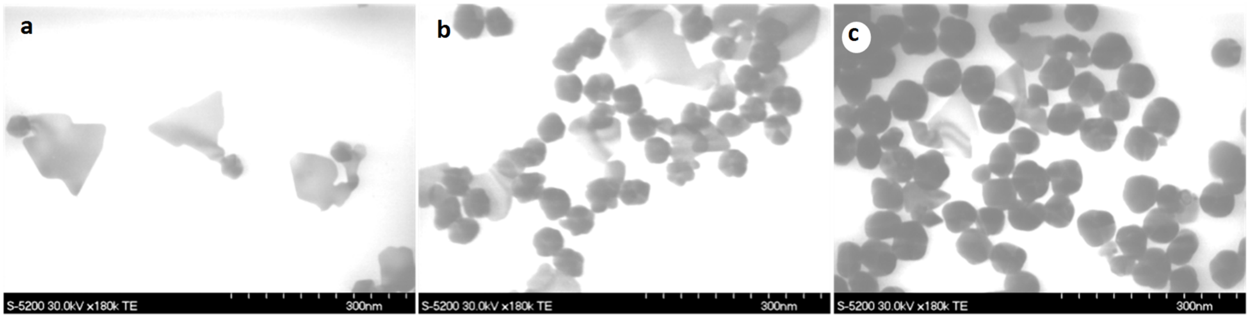

Figure S15. TEM images of bi-AgNP samples prepared using KI with Ag/I molar ratio of **a**) 312:1, **b**) 138:1 and **c**) 6:1.

Iodide interferes with the development of bi-AgNPs even at very low iodide concentrations (sub-µM) corresponding to Ag/I ratio of 312 to 1 (Fig S15a), where rounding of the decahedral seed is observed in addition to the more disturbed growth of the platelet part (Figs. S15b,c). Such effect of iodide is likely due to the fact that AgI does not have the same rock salt structure as AgCl and AgBr, which stabilizes (100) planes. At the same time, K_{sp} of AgI is the lowest of the halides, so the strongest iodide binding is very disruptive for the bi-AgNP regrowth.

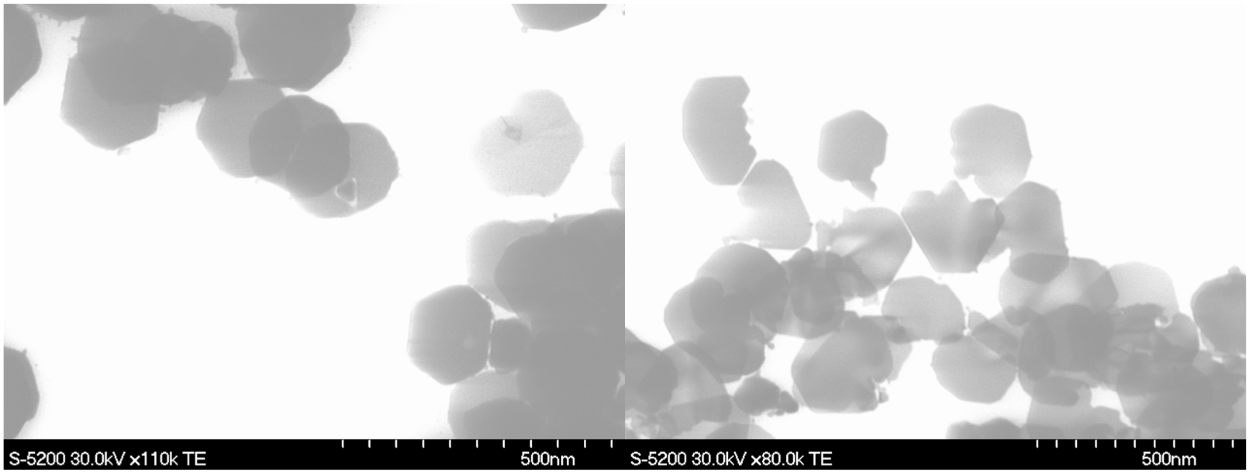

Figure S16. TEM images of bi-AgNPs prepared using silver platlets/prisms as seeds instead of AgDeNPs.

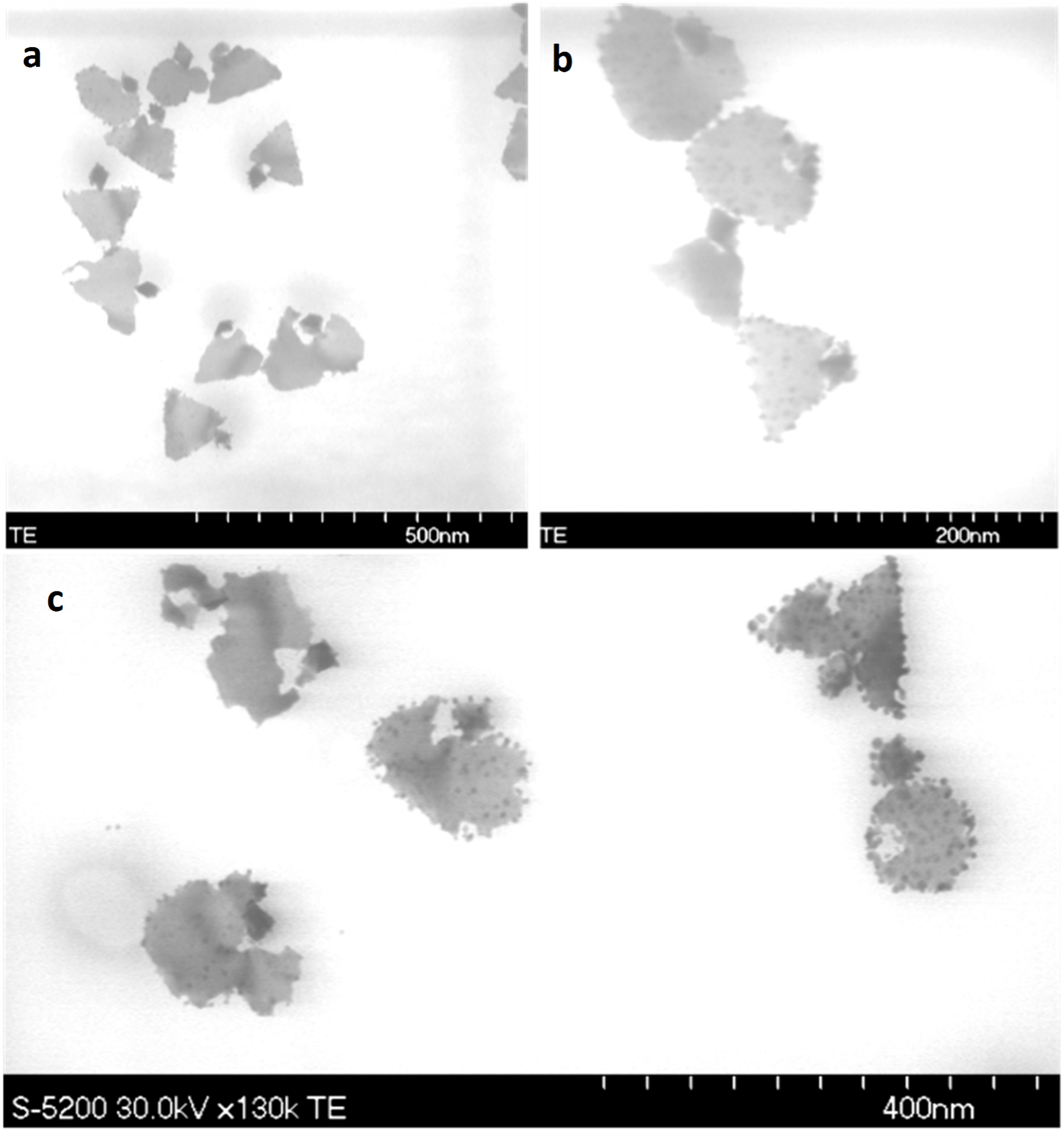

Figure S17. TEM images of bi-AgNP samples that underwent galvanic replacement with tetrachloroauric acid: **a)**, **b)** 5 mol.%; and **c)** 20 mol.% of gold relative to silver in bi-AgNPs.

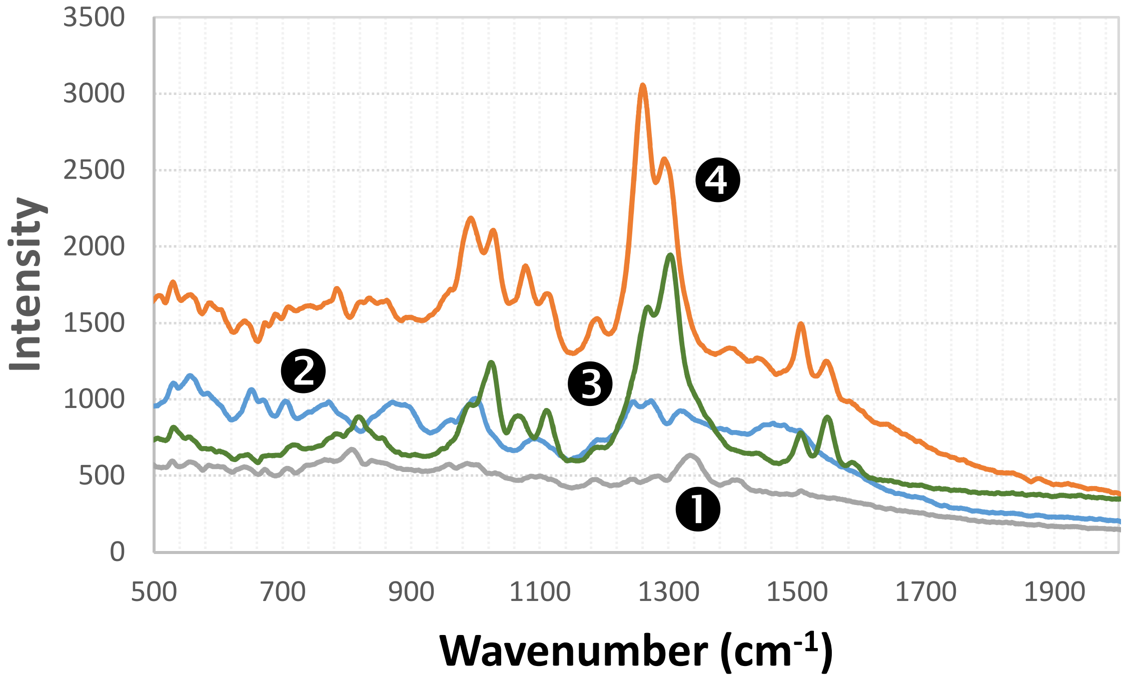

Figure S18. Raman spectra of ❶ bi-AgNP dry layer; ❷ dry layer of decahedral AgNPs; ❸ 1×10^{-16} mol/cm^2 of 5'5-dithiobis(2-nitrobenzoic acid) (DTNB) over the layer of dry bi-AgNPs; and ❹ 2×10^{-16} mol/cm^2 of DTNB over the layer of dry decahedral AgNPs.

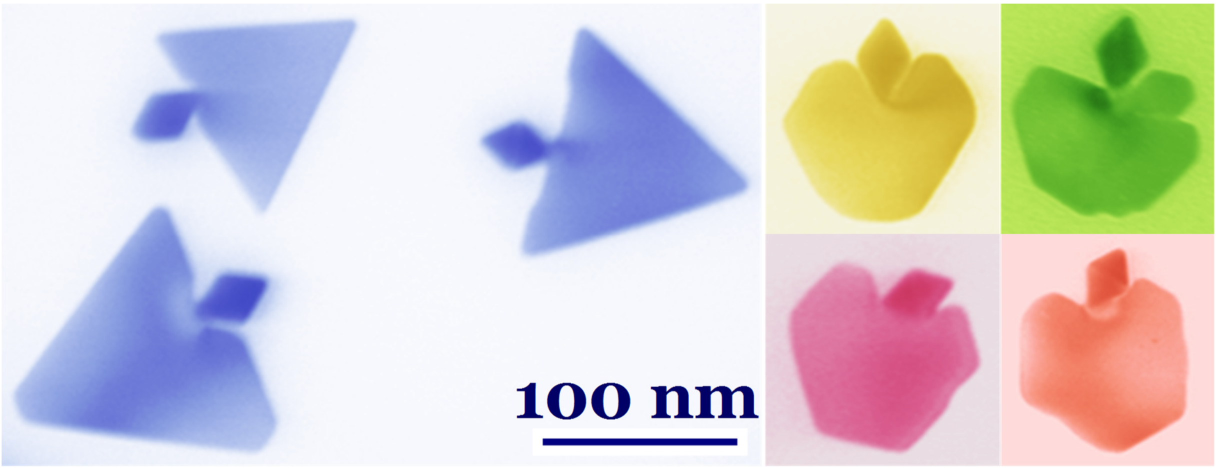

Figure S19. Coloured TEM images of bi-AgNPs resembling fish and apples.

References

[1] Murshid, N., Keogh, D. & Kitaev, V. Optimized synthetic protocols for preparation of versatile plasmonic platform based on silver nanoparticles with pentagonal symmetries. *Part. Part. Syst. Charact.,* **31**, 178 –189 (2014).

[2] Lu, L., Kobayashi, A., Tawa, K. & Ozaki, Y. Silver nanoplates with special shapes: controlled synthesis and their surface plasmon resonance and surface-enhanced Raman scattering properties. *Chem. Mater.,* **18**, 4894 –4901 (2006).
